# Effect of Chemical Bath Deposition Variables on the Properties of Zinc Sulfide Thin Films: A Review

**DOI:** 10.3390/molecules28062780

**Published:** 2023-03-20

**Authors:** Akmal Zaini Arsad, Ahmad Wafi Mahmood Zuhdi, Siti Fazlili Abdullah, Chien Fat Chau, Azrul Ghazali, Ibrahim Ahmad, Wan Syakirah Wan Abdullah

**Affiliations:** 1 Institute of Sustainable Energy, Universiti Tenaga Nasional (UNITEN), Kajang 43000, Malaysia; 2 College of Engineering, Universiti Tenaga Nasional (UNITEN), Kajang 43000, Malaysia; 3 UNITEN R&D, Universiti Tenaga Nasional (UNITEN), Kajang 43000, Malaysia; 4 TNB Renewables Sdn. Bhd., TNB Platinum, No 3, Jalan Bukit Pantai, Kuala Lumpur 591000, Malaysia

**Keywords:** buffer layers, chemical bath deposition, CIGS solar cells, thin films, zinc sulfide

## Abstract

Zinc sulfide (ZnS) thin films prepared using the chemical bath deposition (CBD) method have demonstrated great viability in various uses, encompassing photonics, field emission devices, field emitters, sensors, electroluminescence devices, optoelectronic devices, and are crucial as buffer layers of solar cells. These semiconducting thin films for industrial and research applications are popular among researchers. CBD appears attractive due to its simplicity, cost-effectiveness, low energy consumption, low-temperature compatibility, and superior uniformity for large-area deposition. However, numerous parameters influence the CBD mechanism and the quality of the thin films. This study offers a comprehensive review of the impact of various parameters that can affect different properties of ZnS films grown on CBD. This paper provides an extensive review of the film growth and structural and optical properties of ZnS thin films influenced by various parameters, which include complexing agents, the concentration ratio of the reactants, stirring speed, humidity, deposition temperature, deposition time, pH value, precursor types, and annealing temperature environments. Various studies screened the key influences on the CBD parameters concerning the quality of the resulting films. This work will motivate researchers to provide additional insight into the preparation of ZnS thin films using CBD to optimize this deposition method to its fullest potential.

## 1. Introduction

Zinc sulfide (ZnS) is a metal chalcogenide adhering to the II–VI compound semiconductors that are gaining widespread interest due to their broad range of applications [1,2,3,4]. ZnS is composed of metal and sulfur atoms [5]. Nanostructured metal sulfides provide crystal chemists with a rich subject for study because of their diverse structural characteristics [6]. They are proving to be highly promising materials for the production of a wide range of devices with a wide range of purposes [7,8]. ZnS has n-type conductivity [3] with a band gap semiconductor (3.54 eV) for cubic zinc blende (ZB) and (3.91 eV) for hexagonal wurtzite (WZ) [9,10,11,12]. These values imply that ZnS has a wide band gap [13]. The ZnS optical band gap is wider than cadmium sulfide (CdS), allowing better short-wavelength visible transmittance [14]. For crystalline structures, ZnS has a cubic phase (zinc blende, sphalerite) at room temperature, whereas at higher temperatures, it has a hexagonal phase (wurtzite) [14,15,16]. It shows that ZnS can have two natural phases: cubic and hexagonal [17,18]. At ambient temperature, the exciting binding energy of ZnS (38 MeV) is greater than the thermal energy (25 MeV) allowing for excitonic emission [16]. Moreover, ZnS exhibits other benefits, including non-toxic, earth-abundant, inexpensive, and a refractive index (*n*) of 2.35 at room temperature [11,15,19]. Researchers have also focused on synthesizing ZnS that has been doped with many elements, such as Ni, Fe, Mn, Cu, etc., to modify the chemical and physical properties and generate ZnS for use in a variety of applications, such as achieving enhanced photoluminescence [20]. In addition, Fe: ZnS is a promising candidate for photocatalytic applications [21]. It has been demonstrated that these various dopants produce superior outcomes for other nanomaterials as well [22,23,24,25]. With doped ZnS, impurity compounds contain the Zn lattice site and act as electron and hole traps. Electrons in ZnS are excited from the valence band to the conduction band by absorbing energy equivalent to or higher than their band gap energy [20]. Through doping, it is possible to alter the electrical characteristics (e.g., conductivity) of a material and improve the performance of ZnS [26]. ZnS can also replace cadmium sulfide (CdS) as the buffer layer in copper indium gallium (di) selenide, $\mathrm{Cu}\left(\mathrm{In},\;\mathrm{Ga}\right){\mathrm{Se}}_{2}$ (CIGS) solar cells [15,27]. ZnS is a promising material as the buffer layer because of its excellent chemical and physical properties, notably high visible light transmittance, high photoluminescence, efficient electron mobility, polar surface, excellent charge transport capabilities, etc. [10,26,28]. ZnS’s large band gap also makes it beneficial in other applications, such as UV light emitting diodes (LEDs), solar selective ornamental coatings, flat panel display phosphors, and photocatalysis [19].

ZnS thin films have been synthesized by numerous physical and chemical methods, such as spray pyrolysis [2,3], electrodeposition [15,19], metal-organic vapor phase epitaxy (MOVPE) [29,30], sol-gel [31], magnetron sputtering [32,33], chemical vapor deposition (CVD) [34,35], thermal evaporation [16], molecular beam epitaxy (MBE) [36], atomic layer deposition [37], pulsed-laser deposition (PLD) [14,38], successive ionic layer adsorption and reaction (SILAR), sputtering [4,13], and chemical bath deposition (CBD) technique [1,11,18,39]. Each of the techniques has its advantages and drawbacks. The vapor-phase deposition method necessitates either vacuum conditions or sophisticated equipment [40]. Spray pyrolysis and evaporation require high temperatures making stoichiometry problematic. Some of the films prepared by some of the processes have issues from heat loss and/or loss of optical transmittance, in addition to production method issues that may prevent large-area processing and inexpensive fabrication costs [41]. Among these techniques, the CBD method for synthesizing ZnS thin film is preferable due to its low cost, simple, low-energy consumption, ease of adjusting its parameters, and economically repeatable method that can be employed for low-temperature large-area deposition [1,18,39,42,43,44]. Low-temperature deposition protects metallic substrates against oxidation [45] and is conceivable on a variety of substrates [40]. Additionally, the chemical-solution-deposition-technique-based CBD is capable of producing stoichiometrically fine crystalline phases [41]. In a solution containing the substrates, the CBD approach relies on the controlled release of metal ions (M^2+^) and sulfide (S^2−^) or selenide (Se^2−^) ions. A complexing agent controls M^2+^ discharge in this method. Deposition initiates from nucleation, continues with growth as film thickness rises with time, and ends with film depletion into constituent ions [1]. The CBD-deposited buffer layer is required in thin film solar cell devices to obtain high solar radiation-to-electricity conversion efficiency [46]. Additionally, the CBD method has demonstrated good qualities in ZnS thin film during production. Goudarzi et al. [47] reported that high transmittance of more than 70% had been produced by ZnS thin films grown on a glass substrate using the ammonia-free CBD technique. Nakada and Mizutani [48] reported CBD-ZnS buffer layer on copper indium gallium selenide (CIGS) improved efficiency to 18.1%. Hariskos et al. [49] revealed that CBD-prepared ZnS/CIGS solar cells have a high efficiency of 18.6%. CBD-ZnS might enhance CIGS device efficiency by 20% [49,50].

Despite CBD having several benefits for producing ZnS thin films, other factors must be carefully considered during the thin film process. Controllable factors in the CBD include the Zn source, the S source, the complexing agent, the pH-regulating agent, the bath temperature, the deposition time, etc. [18]. By taking into account all relevant parameters, we can control the reaction rate during CBD deposition so that it proceeds slowly enough to facilitate continuous deposition on substrates, which is the most important factor for a successful CBD deposition procedure [40]. Due to the diversity of parameters influencing the CBD, distinct and inconsistent properties of CBD ZnS films in terms of band gap energy [18,47,51,52,53,54] and optical transmittances [18,47,51,52,53,54] in the ultraviolet–visible region were reported in previous publications. Varying the parameters affected the final chemical and physical properties of the thin films deposited. Discussing ZnS thin film CBD aspects is crucial. With a reliable CBD method to synthesize ZnS thin film, the combination of compounds with superior electrical properties has resulted in the invention of new composite materials that have attracted significant technological interest in recent years. The addition of a second phase can considerably enhance the composite material’s electrical properties [55,56]. ZnS doped with a small percentage of dopants also changes its structural, optical, electrical, and magnetic properties for various purposes [57].

In this work, we give a complete review of the various characteristics of ZnS thin films produced using the CBD method. The deposition of ZnS using CBD is more complex than cadmium sulfide (CdS) [40,58]. This is because ZnS has a low solubility product ($K_{\mathrm{sp}}$): 10^−25^ [14]. Therefore, it can precipitate at low Zn^2+^ and S^2−^ ion concentrations [58]. In particular, the conditions under which ZnS and/or ZnO can deposit simultaneously are much broader [40]. We begin with a brief overview of the investigation into ZnS properties and current research trends in the CBD ZnS thin film development. Next, the CBD method for controlling the parameters for ZnS thin films, including the effect of the complexing agent, concentration ratio [Zn]/[S], stirring, humidity, deposition temperature, deposition time, pH, solution, and Zn precursor, is reviewed. The observed differences in structural, morphological, electronic, optical properties, and chemical bonding results of ZnS thin films formed by the CBD are reviewed. The findings of ZnS thin film with doped ions have attracted considerable interest in the enhancement of the optical, electrical, and structural properties of ZnS thin films are also reviewed. After examining parameter influences on CBD of ZnS thin films, their usefulness, limits, and future possibilities are explored. The objective is to provide suggestions for optimizing the CBD process parameters to produce higher quality ZnS thin films for diverse applications.

## 2. Current Trends in Research and Properties of ZnS Thin Films Utilizing the CBD

This section provides a summary of research trends observed between 1985 and 2023 to provide researchers with an overview of current research trends and potential future study directions in ZnS thin films utilizing the CBD method. Physical, chemical, mechanical, electrical, thermal, and optical film properties were addressed revealing that ZnS films have the potential to be employed in a variety of applications.

### 2.1. Current Research Trends

**Figure 1:**
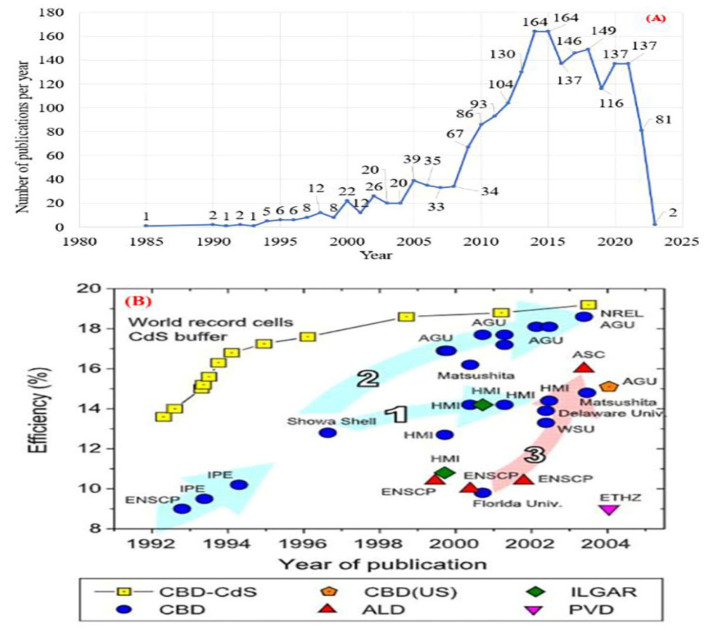
(**A**). Annual trends in publications relating to ZnS thin films prepared by CBD. (**B**) ZnS-based CIGS solar cell buffer layer development. Reproduced with permission from *Thin Solid Films* **2005**, *480–481*, 99–109. Copyright © 2005, Elsevier [49].

The annual trends in ZnS thin film publications prepared by CBD from 1985 to 2023 are shown in Figure 1. The data was extracted from the Scopus database using the keywords “zinc sulfide thin films” and “chemical bath deposition” as highlight keywords. The period of the database search in Scopus was the second week of November 2022. Evaluating Figure 1A from 1985 to 2010, there were a total of 446 publications, while from 2011 to 2023, there were 1560 publications. As history unfolded in 1988, Lokhande et al. [59] proposed the use of CBD for the electrodeposition of ZnS thin films from an acidic bath. In 1992, ENSCP and IPE began developing a CBD ZnS-based buffer layer for CIGS solar cells with 9–10% efficiency [60] (see Figure 1B). In 1996, Showa Shell developed a CIGS cell of 12.8% efficiency with ZnS as its buffer layer. In 1999, Nakada et al. [61] (AGU group) achieved the most efficient CIGS thin film solar cell at the time with a ZnS buffer layer of 16.9% active area efficiency. Afterward, two distinct distribution routes emerged. Showa Shell technique achieved 14–15% efficiency (see arrow 1 in Figure 1B). AGU group’s approach yields 17–19% efficiency (see arrow 2 in Figure 1B) [49]. ASC continued to work on the ZnS-based solar cell buffer and finally achieved 16% efficiency (see arrow 3 in Figure 1B) [49]. Nakada and Mizutani reported in 2002 [48], the CBD-ZnS buffer layer on CIGS increased efficiency to 18.1%. Hariskos et al. [49] recorded that the efficiency of CBD-prepared ZnS/CIGS solar cells is 18.6%. It thus heralded the start of a new era for CBD exploration on ZnS thin films, which helped facilitate new opportunities. As can be demonstrated in Figure 1A, research on CBD ZnS thin film has increased by 77% between 2011 and 2023, a timeframe spanning eleven years. Additionally, the number of papers published in the last eleven years is greater than three times the total number of articles published from 1985 to 2010. It demonstrates that CBD ZnS thin film development is gaining momentum and becoming more widespread.

### 2.2. Properties of Zinc Sulfide Thin Film

ZnS is a noteworthy semiconductor compound of the II–VI group [42,43]. ZnS is chemically and technologically more stable than alternate chalcogenides (such as ZnSe) making it a good host material [9]. ZnS is typically found naturally in two crystalline structures: cubic or zinc blende (ZB) and hexagonal or wurtzite (WZ) [14,15,16]. Both Zn and S have tetrahedral coordination geometry [9]. ZnS exhibits a large band gap at room temperature (300 K) and has attracted considerable interest [44]. The hexagonal phase is a polymorph at high temperatures, while the cubic phase is formed at low temperatures. [43]. Studies [17,62,63] have reported a range of temperatures for crystal structure transition between ZB and WZ phases. As the ZB structure, a = b = c = 5.41 Å when Z = 4, whereas for the WZ phase, a = b = 3.82 Å and c = 6.26 Å when Z = 2 [43]. The minor change in atomic arrangement affects both phases’ physical properties [64]. The reported band gap energy (E_g_) for the ZB and WZ structure are 3.54 eV and 3.91 eV, respectively [9]. ZnS has a greater band gap than ZnO (3.4 eV) [9]. The larger E_g_ enables the layer to transmit more photon energy and exhibit increased light absorption [13]. It is ideal for visible-blind ultraviolet (UV) light-based electronics, including photodetectors and sensors [9]. Zn has a high melting point (1800–1900 °C) that allows researchers to work at high temperatures [43]. Table 1 lists the properties of ZnS. Additionally, nano ZnS has extraordinary chemistry and physical properties, including a high surface-to-volume ratio, quantum size effect, surface and volume effects, macroscopic thermal annealing, enhanced optical absorption, low melting point, and catalysis [9].

The ZnS nanostructure can be used in innovative solar cells, such as a buffer layer for $\mathrm{Cu}\left(\mathrm{In},\;\mathrm{Ga}\right){\mathrm{Se}}_{2}$(CIGS)-based thin film solar cells [49], quantum dot-sensitized solar cells (QDSCs), dye-sensitized solar cells (DSSCs), and organic-inorganic hybrid solar cells [65]. In solar cell applications, the ZnS-based buffer layer is a promising replacement for CBD cadmium sulfide (CdS). Liu and Mao [50] found that CBD-ZnS films have higher transmission in the short wavelength range and a higher band gap (E_g_ = 3.51 eV) than CBD-CdS (E_g_ = 2.41 eV). The ZnS buffer layer is also considered harmless, efficient, and cheap. ZnS has grown as a CIGS buffer layer (Figure 1B) since 1992. Four distinct deposition processes were used: chemical bath deposition (CBD), atomic layer deposition (ALD), physical vapor deposition (PVD), and ion layer gas reaction (ILGAR). To date, CBD has been the most successful approach, described by arrows 1, 2, and 3 in Figure 1B [49].

## 3. Synthesis of ZnS Thin Films Using a Chemical Bath Deposition

Chemical bath deposition (CBD) is an established method for the deposition of thin films. CBD has been employed as a synthesis method for over 140 years [66]. CBD is a chemical process wherein the deposition process is controlled by chemical reactions [43]. CBD has advantages: low-cost, simplicity, uniformity, ease of substrate choice, multi-film runs are conceivable, and controlled growth conditions. Therefore, it is considered to have greater commercial potential than sputtering or thermal evaporation [67].

### 3.1. Basic Experimental Setup for CBD

**Figure 2:**
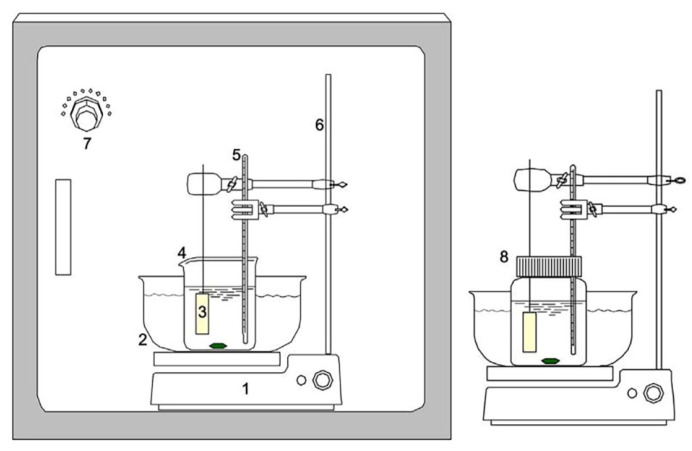
Experimental set-up for CBD with (1) magnetic stirrer, (2) container bath, (3) substrate, (4) beaker, (5) thermometer, (6) clamp stand, (7) relative humidity (RH) controller, and (8) capped vast bottle. Reproduced with permission from *Applied Surface Science* **2014**, *307*, 724–730 [69]. Copyright © 2014, Elsevier.

CBD produces durable, adherent, homogeneous, and rigid films with good reproducibility using a relatively straightforward procedure [43,68]. Figure 2 shows that these methods just require a solution container and a substrate for deposition [69]. The complete deposition system for an open system was placed in an enclosed system with a relative humidity (RH) of 60%, 70%, and 80% [69]. A closed and isolating apparatus is preferred to prevent contamination for the preparation of the sample [43]. Immersing the dipping solution and substrate in a water bath is necessary [43]. A magnetic stirrer is used to properly blend the chemicals in the solutions. Stirring and a thermostatic bath are used to maintain a constant temperature [68]. CBD does not require extremely expensive chemicals processed to a certain level of purity, making it a more affordable process. CBD deposition varies on material composition. During the CBD, researchers may deposit films safely using a fume hood and nontoxic complexing agents.

Substrates can be coated in a single cycle by immersing them in a solution comprising the chalcogenide source, the metal ion, additional acid or base (to adjust the pH of the solution), and a chelating agent [40]. CBD can be used to deposit ZnS solution on a diverse range of substrates, such as silicon [70,71], glass [50], gallium arsenide (GaAs) [70], indium tin oxide (ITO) [72], tin oxide $\left({\mathrm{SnO}}_{2}\right)$ [73], and soda lime glass (SLG) [18,69,74]. Between ZnS, GaAs, and silicon, there is a lattice mismatch of 4.9% and 0.9%, respectively. The glass substrate should be carefully cleaned (washed, scrubbed, degreased, and rinsed) using procedures described in numerous studies [41,75,76,77]. The cleaning technique etches the substrate surface before the deposition to produce nucleation sites, which enhances thin film adherence [41]. CBD chemical bath solution is produced in sufficient volumes to deposit thin coatings on substrates. The reaction solution is thoroughly mixed, and the substrate is vertically clamped into the solution in a beaker covered with synthetic foam to prevent dust or unwanted particles from infiltrating the solution [78]. The solution was obtained in a beaker and left for the appropriate dip periods at deposition temperatures [41].

### 3.2. Basic Principle of CBD

The CBD approach to thin film synthesis is easy and rapid, with deposition at room temperature and normal air pressure, which becomes beneficial from an ecological and economic standpoint [75]. The CBD technique is used to produce ZnS thin films onto glass surfaces [39]. Decomposing a zinc salt, thiourea, and a complexing agent that permits the formation of a soluble Zn^2+^ and S^2−^ species in the solution [39,79]. Zn^2+^ and S^2−^ ions in solution release steadily during deposition, condensing on appropriately mounted substrates to yield ZnS thin films [80].

The CBD solubility principle and ionic product are discussed. CBD’s methodology is dependent on the product’s relative solubility [81]. It is critical to know CBD’s mechanisms in terms of its solubility product ($K_{\mathrm{sp}}$). Considering an extremely sparingly soluble salt (ZnS) in equilibrium with its saturated aqueous solution (when placed in water):

(1) $${\mathrm{ZnS}}_{\left(s\right)}↔{\;\mathrm{Zn}}^{2+}+{\;S}^{2-}$$

Using the law of mass action,

(2) $$K=\frac{\left[{\mathrm{Zn}}^{2+}\right]\left[S^{2-}\right]}{\left[{\mathrm{ZnS}}_{\left(s\right)}\right]}$$

where K is the stability constant,$\;\left[{\mathrm{Zn}}^{2+}\right]\left[S^{2-}\right]$ and $\left[{\mathrm{ZnS}}_{\left(s\right)}\right]$ are concentrations of ${\mathrm{Zn}}^{2+},{\;S}^{2-}$, and ${\mathrm{ZnS}}_{\left(s\right)}$ in the solution, respectively. The concentration of ${\mathrm{ZnS}}_{\left(s\right)}$ (pure solid) is a constant number:

(3) $$\left[{\mathrm{ZnS}}_{\left(s\right)}\right]=\mathrm{constant}=\;K^{\prime }$$

(4) $$K=\frac{\left[{\mathrm{Zn}}^{2+}\right]\left[S^{2-}\right]}{\;K^{\prime }}$$

(5) $${KK}^{\prime }=\left[{\mathrm{Zn}}^{2+}\right]\left[S^{2-}\right]$$

Given that K and K′ are constants, the product of K and K′, labeled K_sp,_ is also a constant. Consequently, Equation (6) yields:

(6) $$K_{\mathrm{sp}}=\left[{\mathrm{Zn}}^{2+}\right]\left[S^{2-}\right]$$

The solubility product (SP) is $K_{\mathrm{sp}}$ and the ionic product (IP) is $\left[{\mathrm{Zn}}^{2+}\right]\left[S^{2-}\right].$ $K_{\mathrm{sp}}$ values for ZnS are 10^−25^ [43,49]. The IP equals the SP when the solution is saturated. Supersaturation, precipitation, and ion nuclei on the substrate and in the solution emerge whenever the IP exceeds the SP [43,81].

The solubility product is affected by three primary factors: temperature, solvent, and particle size [81]. Solubility is the spontaneous interaction between two or more compounds to produce a uniform molecular dispersion. When the solute and solvent are in equilibrium, the solution is considered to be saturated. The equilibrium between a precipitate and its ions in the solution will vary in terms of temperature depending on whether the heat of the solution is endothermic or exothermic [81]. Endothermic and exothermic peaks can be determined by using a differential scanning calorimetry (DSC) curve [82]. Eid et al. [83] had previously reported for the CBD-ZnS powder DSC curve: (a) the initial peak at 100 °C refers to an endothermic reaction, (b) the second peak at 350 °C refers to an exothermic reaction due to the crystallization, and (c) when the temperature reaches 740 °C, the sample undergoes a breakdown or dissociation. Regarding the solvent factor, by adding alcohol or another water-miscible solvent with a low dielectric constant, the solubility of the relatively insoluble material in water is lowered [50]. For the particle size factor, if particle size reduces, it implies that solubility increases [81].

### 3.3. Reaction Mechanism for ZnS Deposition

CBD solids occur in thermodynamically unstable, or “supersaturated” baths. Two reactions produce solid material: (i) homogeneous precipitation (well within the bulk of the solution) and (ii) heterogeneous precipitation (at surfaces substrate or embryogenic reaction on the reaction vessel surface) [84].

**Figure 3:**
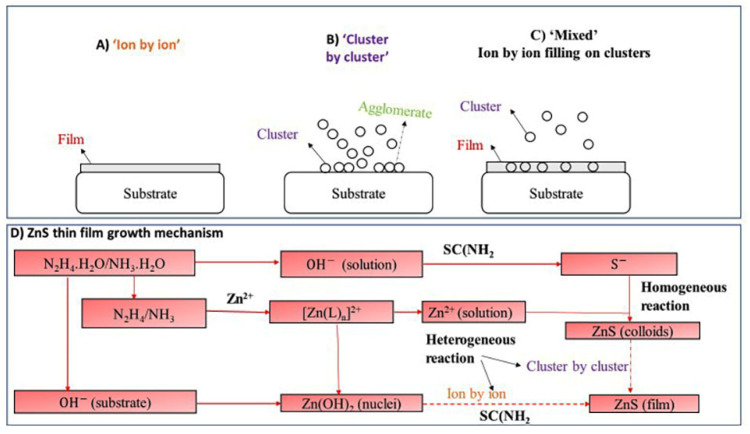
Schematic illustrations of the procedures used to produce ZnS thin films (**A**) ion by ion, (**B**) cluster by cluster, (**C**) mixed; ion-by-ion filling on clusters, and (**D**) ZnS thin film growth mechanism in aqueous alkaline solution. Reproduced with permission from *Materials Science in Semiconductor Processing* **2013**, *16*, 1478–1484. Copyright © 2013, Elsevier [85].

The reaction mechanism for ZnS deposition was discussed in [43,84], which is depicted in Figure 3. Three distinct mechanisms or models regulate the CBD-ZnS thin film deposition. First, ions condense at the interacting surface to produce the film by the ion-by-ion process via a heterogeneous reaction (see Figure 3A). The film has small, compact, and adherent particles. Ion-by-ion growth requires heterogeneous nucleation and nucleus growth [85]. The second is cluster-by-cluster, where surface absorption of colloidal particles pre-formed in solution form particulate films (see Figure 3B). The technique follows a homogenous reaction in which particles from the solution accumulate on a substrate to produce a film [43]. The film has bigger spherical particles, poor compaction and adhesion [85]. The third is both processes (ion-by-ion filling on clusters) may occur and interact, resulting in films with colloidal particles (see Figure 3C). Heterogeneous and homogeneous nucleation determine which process dominates [84]. In this experiment involving both mechanisms, the result is a mixed mechanism [43]. The growth of ZnS films involves a mixed process (see Figure 3D) [85]. In general (aqueous solution), OH^−^ binds through to the substrate, then water-soluble [Zn (L)_n_]^2+^ complex ions react with OH^–^ to free Zn^2+^ to generate Zn(OH) nuclei around which thiourea was bound and hydrolyzed to create the ZnS film. For high-quality ZnS films, it is essential to restrict the cluster-by-cluster mechanism and enhance the ion-by-ion process. Furthermore, it is expected that the ZnS solution formed cluster-by-cluster, whereas the CdS thin film shall form ion-by-ion growth. CBD generated powdery, poor-adhesive, and transparent ZnS films [47].

Based on Liu’s observation [86], they identified an ion-by-ion process as the predominant growth mechanism for ZnS thin films. This reveals that ZnS film growth involves heterogeneous nucleation on the substrate surface and ion-by-ion incorporation. The CBD procedure depends on bath temperature, pH of the reaction solution, reacting species concentration, complexing agents, etc. [85,86]. Great quality (adherent) films are exclusively formed from supersaturated zinc hydroxy solutions depending on the substrate.

### 3.4. Factors Will Affect the CBD for ZnS Thin Film Properties

The CBD method discussed in this work is used to produce ZnS thin films on glass substrates. In the CBD, numerous variables must be controlled, such as the complexing agent, Zn source, sulfur source, stirring speed, humidity, pH, bath temperature, deposition time, precursor types, and annealing (environmental and temperature effect) [18]. These CBD parameters and how they affect different CBD-ZnS thin films are detailed in Table 2. CBD is dependent upon the purity of its elements. All researchers who aim to produce quality research should be mindful of the following factors (depicted in Table 2), since they may have an impact on the significance of their findings. Experimental conditions greatly affect film characteristics [87].

Table 3 shows recent evaluations of ZnS thin films produced with CBD. A variety of CBD experimental procedures for ZnS thin film deposition were demonstrated. CBD-deposited ZnS films have been reported by many researchers. In the literature (see Table 3), the following chemical reagents were employed to prepare ZnS: zinc acetate $\mathrm{Zn}{\left({\mathrm{CH}}_{3}\mathrm{COO}\right)}_{2}$ [88,89], zinc acetate dihydrate $\left(\mathrm{Zn}{\left({\mathrm{CH}}_{3}\mathrm{COO}\right)}_{2}.2H_{2}O\right)$ [11,75,90,91,92,93,94,95], zinc chloride $\left({\mathrm{ZnCl}}_{2}\right)$ [11,76,90,96,97,98], zinc nitrate $\left(\mathrm{Zn}{\left({\mathrm{NO}}_{3}\right)}_{2}\right)$ [99], zinc nitrate hexahydrate $\left(\mathrm{Zn}{\left({\mathrm{NO}}_{3}\right)}_{2.}6H_{2}O\right)$ [90], zinc sulfate $\left({\mathrm{ZnSO}}_{4}\right)\;$[11,77,78,85,100,101,102,103], and zinc sulfate heptahydrate $\left({\mathrm{ZnSO}}_{4}.7H_{2}O\right)\;$[21,58,69,90,104,105,106,107,108,109,110] as a source of Zn^2+^ ions; sodium sulfide $\left({\mathrm{Na}}_{2}S\right)$ [94], thioacetamide $\left(C_{2}H_{5}\mathrm{NS}\right)$ [88,90], and thiourea $\left(\mathrm{SC}{\left({\mathrm{NH}}_{2}\right)}_{2}\right)$ [11,21,58,69,75,76,77,78,85,89,92,93,95,96,97,98,99,100,101,102,103,104,105,106,107,108,109,110,111] as a source of S^2−^ ions; and acetic acid (CH₃COOH) [88], ammonia $\left({\mathrm{NH}}_{3}\right)$ [11,77,78,85,89,93,96,97,102,103,107,109] ammonium chloride $\left({\mathrm{NH}}_{4}\mathrm{Cl}\right)$ [91], ammonia hydroxide $\left({\mathrm{NH}}_{4}\mathrm{OH}\right)$ [11,21,58,91,101,105,106,108], ammonium nitrate $\left({\mathrm{NH}}_{4}{\mathrm{NO}}_{3}\right)$ [76,98,99] ethylenediamine tetra-acetate (EDTA) $\left(C_{10}H_{16}N_{2}O_{8}\right)$ [112], hydrazine $\left(N_{2}H_{4}\right)$ [85,100,105,106,107], hydrazine monohydrate $\left(N_{2}H_{4}.H_{2}O\right)$ [21,69,110], triethanolamine $\left(C_{6}H_{15}{\mathrm{NO}}_{3}\right)$ [94], tri-sodium citrate $\left({\mathrm{Na}}_{3}-\mathrm{citrate}:{\;\mathrm{Na}}_{3}C_{6}H_{5}O_{7}\right)$ [18,75,91,92,96,109,112], trisodium citrate dehydrate $({\mathrm{Na}}_{3}C_{6}H_{5}O_{7}.H_{2}O)\;$[95,104], tartaric acid $\left(C_{4}H_{6}O_{6}\right)$ [95], and urea $\left({\mathrm{CH}}_{4}N_{2}O\right)$ [90] as a complexing agent. ${\mathrm{Na}}_{3}-\mathrm{citrate}$ [18,75,91,92,96,112] and ethylenediamine tetra-acetate (EDTA) $\left(C_{10}H_{16}N_{2}O_{8}\right)$ [112] were utilized as non-toxic complexing agents. By adding ammonia (${\mathrm{NH}}_{4}\mathrm{OH}$), the pH regulator of an alkaline medium and potassium hydroxide (KOH) were altered [18,69,75,85,96,100], [76,98] whereas the acidic solution was adjusted by hydrochloric acid (HCl) [90] and sulfuric acid $\left(H_{2}{\mathrm{SO}}_{4}\right)$ [94].

Depending on the literature, the ZnS film had varying outcomes. Tec-Yam et al. [76] mentioned the quality of ZnS films concerning their morphologies and optical and structural properties. Inconsistency in the properties of CBD ZnS films, such as thickness, transmittance, and band gap energy, is mostly attributable to the varied compositions and microstructures of the film that resulted from the CBD-controlled parameters (see Table 3). Optical measurements of ZnS thin films were typically measured 300–800 nm [18,75,76,91,96,100,101,112], and the energy band gap was computed using appropriate relations. The high transmittance and low reflection of ZnS film growth on glass substrates (see Table 3) indicate that they are an excellent candidate for antireflective applications. To provide a concise summary, Section 3.4.1, Section 3.4.2, Section 3.4.3, Section 3.4.4, Section 3.4.5, Section 3.4.6, Section 3.4.7, Section 3.4.8 and Section 3.4.9 discuss all the parameters and their potential effects on CBD-ZnS film properties. ZnS thin films can be buffer layers for $\mathrm{Cu}\left(\mathrm{In},\;\mathrm{Ga}\right){\mathrm{Se}}_{2}$ solar cells with optimized CBD parameters [58,76].

**Table 3:** A recent study of CBD-prepared ZnS thin films’ parameters and properties.

Ref	Years	Bath Composition & Molarity	Substrate	pH	Complexing Agent	Deposition of Temperature (°C)	Deposition of Time	Stirring Speed (rpm)	Annealing Temperature (°C)	Annealing Environment	Properties Remarks		
											Thickness (nm)	Transmittance (%)	E_g_ (eV)
[75]	2012	$\mathrm{Zn}{\left({\mathrm{CH}}_{3}\mathrm{COO}\right)}_{2}.2H_{2}O$ $\;(0.2\;M),\;\mathrm{SC}{\left({\mathrm{NH}}_{2}\right)}_{2}$ $\;(0.4\;M),\;{\mathrm{Na}}_{3}-\mathrm{citrate}$ $\;(0–0.2\;M),\;{\mathrm{NH}}_{4}\mathrm{OH}$	Glass	10.0	${\mathrm{Na}}_{3}-\mathrm{citrate}$	80	4 h	-	-	-	70–140	75–85%	3.73–3.80
[76]	2012	${\mathrm{ZnCl}}_{2}$$\;(0.02\;M),\;\mathrm{SC}{\left({\mathrm{NH}}_{2}\right)}_{2}$$(0.2\;M),\;{\mathrm{NH}}_{4}\mathrm{OH}$ (1.5 M), KOH (0.8–1.4 M)	Glass	10.0–12.0	${\mathrm{NH}}_{4}\mathrm{OH}$	90	60–120 min	-	-	-	60–135	>85%	3.68–3.89
[58]	2012	${\mathrm{ZnSO}}_{4}.7H_{2}O$$\;(0.03\;M),\;\mathrm{SC}{\left({\mathrm{NH}}_{2}\right)}_{2}$$\;(0.1–0.5\;M),\;{\mathrm{NH}}_{4}\mathrm{OH}$ (0.3–3 M)	ITO	-	${\mathrm{NH}}_{4}\mathrm{OH}$	80	-	300	120 °C for 20 min	-	40–90	>80%	3.73–3.79
[112]	2012	$\mathrm{Zn}{\left({\mathrm{CH}}_{3}\mathrm{COO}\right)}_{2}.2H_{2}O$$\;(0.2M),\;{\mathrm{Na}}_{3}-\mathrm{citrate}$ (0.2M), EDTA (0.4 M)	Glass	10.0	${\mathrm{Na}}_{3}-\mathrm{citrate}$, EDTA	80	4 h	-	-	-	>100	70–85%	3.84–3.94
[100]	2012	${\mathrm{ZnSO}}_{4}$ $,\;\mathrm{SC}{\left({\mathrm{NH}}_{2}\right)}_{2}$ $,\;N_{2}H_{4}$ $,\;{\mathrm{NH}}_{4}\mathrm{OH}$	Glass, silicon	11.0	${\mathrm{NH}}_{4}\mathrm{OH}$ $,\;N_{2}H_{4}$	80	10–60 min	-	-	-	33.8–78.8	>80%	3.83–3.85
[85]	2013	${\mathrm{ZnSO}}_{4}.7H_{2}O$ $,\;\mathrm{SC}{\left({\mathrm{NH}}_{2}\right)}_{2}$ $,\;{\mathrm{NH}}_{3}$ $,\;N_{2}H_{4}$	SLG	8.3–10.6	${\mathrm{NH}}_{3}$ $,\;N_{2}H_{4}$	70	2 h	-	200 °C for 1 h	-	80	>70%	3.76–3.87
[105]	2013	${\mathrm{ZnSO}}_{4}.7H_{2}O,$ $\mathrm{SC}{\left({\mathrm{NH}}_{2}\right)}_{2},$ $N_{2}H_{4}$ $,\;{\mathrm{NH}}_{4}\mathrm{OH}$	Glass	9.0–11.0	${\mathrm{NH}}_{4}\mathrm{OH}$ $,\;N_{2}H_{4}$	80	1 h	-	550 °C for 2 h	-	54–122	75–80%	4.0–4.2
[101]	2013	${\mathrm{ZnSO}}_{4}\;$$(0.025\;M),\;\mathrm{SC}{\left({\mathrm{NH}}_{2}\right)}_{2}$ $(0.27\;M),\;{\mathrm{NH}}_{4}\mathrm{OH}$ (2.9 M)	Glass	-	${\mathrm{NH}}_{4}\mathrm{OH}$	75–95	2 h	-	200 °C	-	73–200	78%	-
[90]	2013	${\mathrm{ZnSO}}_{4}.7H_{2}O$$\;(0.02\;M),\;{\mathrm{ZnCl}}_{2}$ $(0.02\;M),\;\mathrm{Zn}{\left({\mathrm{CH}}_{3}\mathrm{COO}\right)}_{2}.2H_{2}O$$\;(0.02\;M),\;\mathrm{Zn}{\left({\mathrm{NO}}_{3}\right)}_{2.}6H_{2}O$$\;(0.02\;M),\;C_{2}H_{5}\mathrm{NS}\;\left(0.02\;M\right),$ urea (0.5 M)	Glass	5.9–6.1	${\mathrm{CH}}_{4}N_{2}O$	85	4 h	-	500 °C	$\mathrm{Ar}/H_{2}S\;$(5%)	133–175	>70%	3.66–3.83
[91]	2013	$\mathrm{Zn}{\left({\mathrm{CH}}_{3}\mathrm{COO}\right)}_{2}.2H_{2}O$$\;(0.15\;M),\;\mathrm{SC}{\left({\mathrm{NH}}_{2}\right)}_{2}$ $(1\;M),\;{\mathrm{NH}}_{4}\mathrm{OH}$$,\;{\mathrm{NH}}_{4}\mathrm{Cl}$$,\;\;{\mathrm{Na}}_{3}-\mathrm{citrate}$ (0.5M)	Glass, SiO_2_	10.0	${\mathrm{Na}}_{3}-\mathrm{citrate}$ $,\;{\mathrm{NH}}_{4}\mathrm{OH}$ $,\;{\mathrm{NH}}_{4}\mathrm{Cl}$	79–80	30–90 min	-	-	-	60	>80%	3.62
[18]	2013	${\mathrm{ZnSO}}_{4},\;\mathrm{SC}{\left({\mathrm{NH}}_{2}\right)}_{2},$ ${\mathrm{NH}}_{3},$ ${\mathrm{Na}}_{3}-\mathrm{citrate}$	SLG	10.0–12.0	${\mathrm{Na}}_{3}-\mathrm{citrate}$	75–85	20–80 min	-	400 °C for 1 h	$N_{2}$	27–301	70.8–87.8%	3.881–3.980
[77]	2014	${\mathrm{ZnSO}}_{4}$ $,\;\mathrm{SC}{\left({\mathrm{NH}}_{2}\right)}_{2}$ $,\;{\mathrm{NH}}_{3}$	Glass	10.0	${\mathrm{NH}}_{3}$	80	1 h	-	400 °C for 1 h 30 min	-	60	-	3.099–3.215
[106]	2014	${\mathrm{ZnSO}}_{4}.7H_{2}O,$ $\mathrm{SC}{\left({\mathrm{NH}}_{2}\right)}_{2},$ ${\mathrm{NH}}_{4}\mathrm{OH}$ $,\;N_{2}H_{4}$	SLG	9.8–10.6	$N_{2}H_{4}$	50–90	1.5–2.5 h	-	-	-	40–160	>80%	3.93–4.06
[69]	2014	${\mathrm{ZnSO}}_{4}.7H_{2}O$ $\;(0.15\;M),\;\mathrm{SC}{\left({\mathrm{NH}}_{2}\right)}_{2}$ $\;(0.9\;M),\;N_{2}H_{4}.H_{2}O$	SLG	9.7	$N_{2}H_{4}.H_{2}O$	80	20–120 min	650	-	-	50	77%	3.78–3.96
[102]	2015	${\mathrm{ZnSO}}_{4}$ $(0.010\;M),\;\mathrm{SC}{\left({\mathrm{NH}}_{2}\right)}_{2}$$\;(0.8\;M),\;{\mathrm{NH}}_{3}$ (0.07 M)	SLG	10.0	${\mathrm{NH}}_{3}$	90	-	-	100–300 °C	-	-	70–80%	3.82–3.89
[78]	2015	${\mathrm{ZnSO}}_{4}$ $(0.025\;M),\;\mathrm{SC}{\left({\mathrm{NH}}_{2}\right)}_{2}$$\;(2.9\;M),\;{\mathrm{NH}}_{4}\mathrm{OH}$ (0.2 M)	Glass	-	${\mathrm{NH}}_{4}\mathrm{OH}$	70–90	-	-	-	-	110	90–80%	3.57–3.78
[11]	2018	${\mathrm{ZnSO}}_{4}$$\;(0.15\;M),\;\mathrm{Zn}{\left({\mathrm{CH}}_{3}\mathrm{COO}\right)}_{2}.2H_{2}O$$\;(0.15\;M),\;{\mathrm{ZnCl}}_{2}$ $(0.15\;M),\;\mathrm{SC}{\left({\mathrm{NH}}_{2}\right)}_{2}$ (0.6 M), $\;{\mathrm{NH}}_{3}$ (7.5 M)	Glass	-	${\mathrm{NH}}_{4}\mathrm{OH}$	70–90	-	-	250 °C for 10 min	-	30–90	-	3.40–3.49
[104]	2018	${\mathrm{ZnSO}}_{4}.7H_{2}O$ $,\;\mathrm{SC}{\left({\mathrm{NH}}_{2}\right)}_{2}$ $,\;{\mathrm{Na}}_{3}C_{6}H_{5}O_{7}.H_{2}O$	Glass	10.7	${\mathrm{Na}}_{3}C_{6}H_{5}O_{7}.H_{2}O$	70	2–6 h	-	-	-	68–134	>80%	3.69–3.77
[99]	2018	$\mathrm{Zn}{\left({\mathrm{NO}}_{3}\right)}_{2},\;\mathrm{SC}{\left({\mathrm{NH}}_{2}\right)}_{2},\;{\mathrm{NH}}_{3}$	Glass	12	${\mathrm{NH}}_{3}$	85	1 h	-	-	-	-	-	3.36–3.69
[103]	2019	${\mathrm{ZnSO}}_{4}$ $,\;\mathrm{SC}{\left({\mathrm{NH}}_{2}\right)}_{2}$ $,\;{\mathrm{NH}}_{3}$	Glass	-	${\mathrm{NH}}_{3}$	65–80	20–50 min	-	-	-	70–160	93.7–99%	3.97–4.05
[96]	2019	${\mathrm{ZnCl}}_{2}$ $,\;\mathrm{SC}{\left({\mathrm{NH}}_{2}\right)}_{2}$ $,\;{\mathrm{NH}}_{3}$	Glass	11.0	${\mathrm{Na}}_{3}-\mathrm{citrate}$	80	60–150 min	-	500 °C for 2 h	-	-	69–81%	3.87–4.03
[95]	2019	$\mathrm{Zn}{\left({\mathrm{CH}}_{3}\mathrm{COO}\right)}_{2}.2H_{2}O\;\left(0.063\;M\right),\;\mathrm{SC}{\left({\mathrm{NH}}_{2}\right)}_{2}\;\left(0.063\;M\right)\;,$ $\mathrm{SC}{\left({\mathrm{NH}}_{2}\right)}_{2},$ ${\mathrm{Na}}_{3}C_{6}H_{5}O_{7}.H_{2}O$ $\;(0.013\;M),\;C_{4}H_{6}O_{6}\;\left(0.047\;M\right)$	Glass	-	${\mathrm{Na}}_{3}C_{6}H_{5}O_{7}.H_{2}O,{\;C}_{4}H_{6}O_{6}$	70	30 min	180 (2 h)	500 °C for 1 h	Sulfur	-	50–80%	-
[92]	2020	$\mathrm{Zn}{\left({\mathrm{CH}}_{3}\mathrm{COO}\right)}_{2}.2H_{2}O,$ $\mathrm{SC}{\left({\mathrm{NH}}_{2}\right)}_{2},$ ${\mathrm{Na}}_{3}C_{6}H_{5}O_{7}$	Glass	9.0–10.6	${\mathrm{Na}}_{3}C_{6}H_{5}O_{7}$	80	3 h	-	-	-	21–199	>70%	3.78–4.00
[107]	2020	${\mathrm{ZnSO}}_{4}.7H_{2}O$$\;(0.019\;M),\;\mathrm{SC}{\left({\mathrm{NH}}_{2}\right)}_{2}$ $(0.17\;M),\;{\mathrm{NH}}_{3},$$N_{2}H_{4}$	SLG	9.0–10.8	${\mathrm{NH}}_{3}$ $,\;N_{2}H_{4}$	80	0–2 h	-	200 °C for 10 min	-	37–75	70–80%	3.64–3.75
[108]	2021	${\mathrm{ZnSO}}_{4}.7H_{2}O$ $\;(0.019\;M),\;\mathrm{SC}{\left({\mathrm{NH}}_{2}\right)}_{2}$ $\;(0.17\;M),\;{\mathrm{NH}}_{4}\mathrm{OH}$	Glass	9.8	${\mathrm{NH}}_{4}\mathrm{OH}$	60	45 min	-	100–300 °C for 1 h	-	40–130	76%	3.93–3.98
[94]	2021	$\mathrm{Zn}{\left({\mathrm{CH}}_{3}\mathrm{COO}\right)}_{2}\;.2H_{2}O\;\left(0.1-0.4\;M\right),{\;N}_{2}S\;\left(0.1\;M\right),$ $\;C_{6}H_{15}{\mathrm{NO}}_{3}$	Glass	5	$C_{6}H_{15}{\mathrm{NO}}_{3}$	80	90 min	-	-	-	-	-	2.6–3.5
[89]	2021	$\mathrm{Zn}{\left({\mathrm{CH}}_{3}\mathrm{COO}\right)}_{2}\left(0.1M\right),\;\mathrm{SC}{\left({\mathrm{NH}}_{2}\right)}_{2}\;\left(0.3\;M\right),{\;\mathrm{NH}}_{4}$ (5 M)	Glass, quartz	-	${\mathrm{NH}}_{4}$	90 (aged 1 h)	60–70 min	-	350 °C for 20 min	Nitrogen	239–590	>70%	3.62–3.68
[110]	2021	${\mathrm{ZnSO}}_{4}.7H_{2}O\;\left(0.035\;M\right),\;\mathrm{SC}{\left({\mathrm{NH}}_{2}\right)}_{2}\;\left(0.035\;M\right)$, ${\mathrm{NH}}_{4}\mathrm{OH}\;\left(1.1\;M\right)$, $N_{2}H_{4}$.$H_{2}O$ (3 M)	Glass	-	${\mathrm{NH}}_{4}\mathrm{OH},{\;N}_{2}H_{4}$ $.H_{2}O$	80	1 h	-	-	-	-	70%	3.70
[97]	2021	${\mathrm{ZnCl}}_{2}$ $,\;\mathrm{SC}{\left({\mathrm{NH}}_{2}\right)}_{2}$ $,\;{\mathrm{NH}}_{3}$	Glass	9	${\mathrm{NH}}_{3}$	-	1 h	-	150–300 °C	-	450	77.32–79.43%	3.34–3.45
[98]	2021	${\mathrm{ZnCl}}_{2}$ $\mathrm{SC}{\left({\mathrm{NH}}_{2}\right)}_{2}$ $,\;{\mathrm{NH}}_{4}{\mathrm{NO}}_{3}$	Glass	11.5–12.5	${\mathrm{NH}}_{4}{\mathrm{NO}}_{3}$	40–60	-	-	-	-	-	60–95%	3.72
[88]	2022	$\mathrm{Zn}{\left({\mathrm{CH}}_{3}\mathrm{COO}\right)}_{2}\left(1.0\;M\right),\;C_{2}H_{5}\mathrm{NS}\;\left(1.0\;M\right),\;\mathrm{CH}₃\mathrm{COOH}$	Glass	6.5–7.0	CH₃COOH	-	10–60 min	-	-	-	40–109	60–90%	3.60–3.85
[21]	2022	${\mathrm{ZnSO}}_{4}.7H_{2}O$$,\;\mathrm{SC}{\left({\mathrm{NH}}_{2}\right)}_{2},$ ${\mathrm{NH}}_{4}\mathrm{OH},{\;N}_{2}H_{4}H_{2}O$	Glass	-	${\mathrm{NH}}_{4}\mathrm{OH},{\;N}_{2}H_{4}H_{2}O$	80	60 min	-	-	-	-	70%	3.7
[109]	2022	${\mathrm{ZnSO}}_{4}.7H_{2}O$ (0.1 M), $\mathrm{SC}{\left({\mathrm{NH}}_{2}\right)}_{2}\;\left(0.4\;M-0.6\;M\right)\;$	Glass		${\mathrm{NH}}_{4},\;C_{6}H_{15}{\mathrm{NO}}_{3},{\;\mathrm{Na}}_{3}-\mathrm{citrate}.\;$	75	90 min	-	-	-	180–121	>70%	3.5–3.75
[93]	2022	$\mathrm{Zn}{\left({\mathrm{CH}}_{3}\mathrm{COO}\right)}_{2}.2H_{2}O$$\;(1\;M),\;\mathrm{SC}{\left({\mathrm{NH}}_{2}\right)}_{2}$ $(1\;M),\;{\mathrm{NH}}_{3}$	SLG	5	${\mathrm{NH}}_{3}$	-	-	-	500 °C for 30 min	vacuum	90.44–101.32	15.82–75.782%	4.15–4.56

Ar = argon, CBD = chemical bath deposition, E_g_ = band gap energy, $H_{2}$ = hydrogen, ITO = indium tin oxide, $N_{2}$ = nitrogen, SLG = soda lime glass, SiO_2_ = silicon dioxide, and ZnS = zinc sulfide. The complexing agent was utilized including acetic acid (CH₃COOH) [88], ammonia $\left({\mathrm{NH}}_{3}\right)$ [11,77,78,85,89,93,96,97,102,103,107], ammonium chloride $\left({\mathrm{NH}}_{4}\mathrm{Cl}\right)$ [91], ammonia hydroxide $\left({\mathrm{NH}}_{4}\mathrm{OH}\right)$ [11,21,58,91,101,105,106,108,110], ammonium nitrate $\left({\mathrm{NH}}_{4}{\mathrm{NO}}_{3}\right)$ [76,98], ethylenediamine tetra-acetate (EDTA: $C_{10}H_{16}N_{2}O_{8})$ [112], hydrazine $\left(N_{2}H_{4}\right)$ [85,100,105,106,107], hydrazine monohydrate $\left(N_{2}H_{4}.H_{2}O\right)$ [21,69,110], triethanolamine (TEA: $C_{6}H_{15}{\mathrm{NO}}_{3})$ [94,109], tri-sodium citrate $({\mathrm{Na}}_{3}-\mathrm{citrate}:$ ${\mathrm{Na}}_{3}C_{6}H_{5}O_{7})$ [18,75,91,92,96,109,112], trisodium citrate dehydrate $({\mathrm{Na}}_{3}C_{6}H_{5}O_{7}.H_{2}O)\;$[95,104], tartaric acid $\left(C_{4}H_{6}O_{6}\right),$ [95] and urea (${\mathrm{CH}}_{4}N_{2}O$) [90]. The pH regulator of an alkaline solution was adjusted by adding ammonia (${\mathrm{NH}}_{4}\mathrm{OH}$) [18,69,75,85,96,100] and potassium hydroxide (KOH) [76], and the acidic solution was adjusted by hydrochloric acid (HCl) [90]. The sulfur sources utilized included sodium sulfide ($N_{2}S)$ [94], thioacetamide ($C_{2}H_{5}\mathrm{NS}$) [88,90], and thiourea ($\mathrm{SC}{\left({\mathrm{NH}}_{2}\right)}_{2}$) [11,21,58,69,75,76,77,78,85,89,92,93,95,96,97,98,100,101,102,103,104,105,106,107,108,109,110]. The zinc sources utilized included zinc acetate ($\mathrm{Zn}{\left({\mathrm{CH}}_{3}\mathrm{COO}\right)}_{2})$ [88,89], zinc acetate dihydrate $\left(\mathrm{Zn}{\left({\mathrm{CH}}_{3}\mathrm{COO}\right)}_{2}.2H_{2}O\right)$ [11,75,90,91,92,93,94,95], zinc chloride (${\mathrm{ZnCl}}_{2}$) [11,76,90,96,98], zinc nitrate, zinc nitrate hexahydrate $\left(\mathrm{Zn}{\left({\mathrm{NO}}_{3}\right)}_{2.}6H_{2}O\right)$ [90], zinc sulfate (${\mathrm{ZnSO}}_{4}$) [11,77,78,85,100,101,102,103], and zinc sulfate heptahydrate (${\mathrm{ZnSO}}_{4}.7H_{2}O)\;$[21,58,69,90,104,105,106,107,108,109,110].

#### 3.4.1. Influence of Complexing Agents

The CBD approach relies on the controlled precipitation of a deposit from a solution onto a substrate [105]. The complexing agent is a crucial thin film deposition condition for CBD synthesis-based ZnS thin films. A complexing agent binds metallic ions to prevent homogenous precipitation. The created metal complex hydrolyzes slowly to produce positive ions. ZnS thin films are deposited when Zn^2+^ and S^2−^ ions exceed ZnS solubility. A homogeneous or heterogeneous deposition is possible. The faster homogenous process precipitates powdery ZnS particles in large quantities on the substrate. For homogeneity reduction, metal complexes must form. In the heterogeneous process, Zn^2+^ and S^2-^ ions are slowly released into liquid and condense on the substrate [12]. The complexing agent is particularly beneficial in preventing precipitation on a solid surface and preventing powder production in the bath solution.

To deposit ZnS using CBD is challenging due to its very low value of $K_{\mathrm{sp}}$ = 10^−24.7^ [75]. To generate homogenous ZnS thin films, numerous researchers have consequently utilized complexing agents to modulate Zn^2^+ ion content during deposition [52,75,85]. The complexing agent aids in thin film formation [85] and affects the morphology of the ZnS layer [52]. Furthermore, a complexing agent in a solution expands the duration of the deposition bath and improves film adhesion on glass substrates [19]. One or even more complex agents may be employed in CBD-ZnS thin films [52]. During thin film deposition, researchers utilize ammonia $\left({\mathrm{NH}}_{3}\right)$ [39,113], hydrazine hydrate $\left(N_{2}H_{4}.H_{2}O\right)$, disodium ethylene diamine tetra-acetate, tartaric acid, nitrilotriacetic acid [114], etc. for the complexing agent [19]. Hydrazine hydrate (HH), tri-ethanolamine (TEA), and trisodium-citrate (TSC) form stable compounds with zinc ions, inhibiting zinc hydroxide and zinc oxide production [115].

Mushtaq et al. [93] reported the CBD of ZnS onto glass substrates with ammonia and without ammonia. No contaminant peaks were found for the films with the addition of ammonia. Compared to the films without ammonia solution, the film with ammonia solution had a smooth surface with few pores. The addition of ammonia to ZnS films increased the transmittance from 15.82% to 75.82%. ZnS films with ammonia demonstrated smaller energy band gap values than those without ammonia. All films had band gaps between 4.15 and 4.56 eV.

Goudarzi et al. [88] used the microwave-assisted chemical bath deposition (MA-CBD) method to prepare the ZnS film in a short period without an ammonia solution. Acetic acid as a complexing agent was utilized. XRD revealed the morphology was highly crystalline and cubic. The FE-SEM result showed homogeneous, compact, small particles in the films without cracks or pinholes. Hydrazine is a common additive complexing agent for ZnS deposited via CBD. It increases uniformity, improves homogeneity, and improves growth rate as well as interfacial adherence of the ZnS thin films [50,75,80]. Hydrazine also functions as a bridge-forming ligand and enhances surface binding [39,40]. Hydrazine complexes offer a less steric barrier to the sulfide ion due to having a lower coordination number [40]. Complexing agents, such as ammonia and hydrazine, were popular for CBD to prepare ZnS thin films [40,105,115]. Dona and Herrero [80], Wei et al. [85], and Liu et al. [86] demonstrated CBD-complexing agents for ZnS thin films using ${\mathrm{NH}}_{3}$ and $N_{2}H_{4}$.

**Figure 4:**
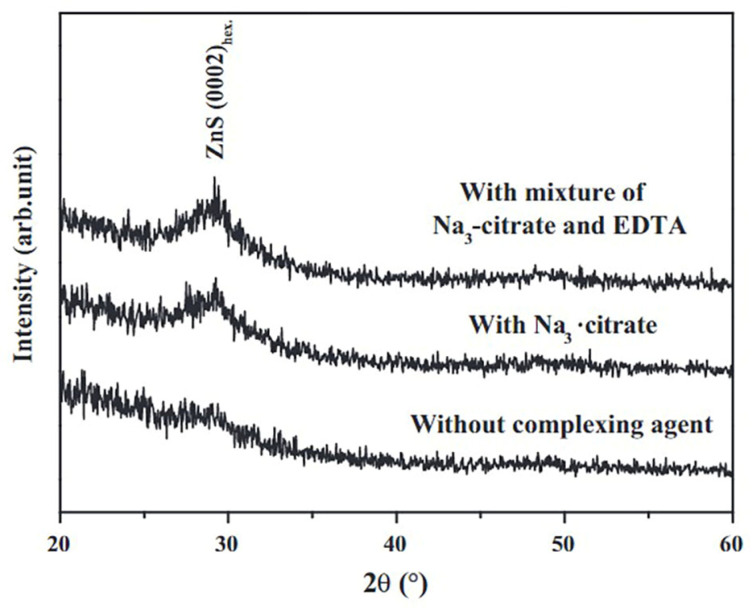
XRD examination (PDF card: 65-9585) of ZnS thin films without complexing agents, with ${\mathrm{Na}}_{3}-\mathrm{citrate}$, and combined ${\mathrm{Na}}_{3}-\mathrm{citrate}$ and EDTA. Reproduced with permission from *Journal of Alloys and Compounds* **2012**, *526*, 25–30. Copyright © 2012, Elsevier [112].

However, hydrazine hydrate is a material that is extremely combustible, carcinogenic, and poisonous [80]. Several researchers have studied ZnS thin film deposition utilizing less harmful complexing agents, such as ${\mathrm{Na}}_{3}-\mathrm{citrate}$ [18,75,116], EDTA [47], and tartaric acid [1]. All of these complex compounds were developed to replace hydrazine hydrate with non-toxic chemicals. Shin et al. [112] evaluated the impact of complexing agents on ZnS thin film’s structural, chemical, compositional, morphological, optical, and growth properties. Zinc acetate, thiourea, ${\mathrm{Na}}_{3}-\mathrm{citrate}$, and ${\mathrm{Na}}_{3}-\mathrm{citrate}$/EDTA were utilized for the production of ZnS thin films on glass substrates. CBD-ZnS thin films were prepared (a) without the complexing agent, (b) with ${\mathrm{Na}}_{3}-\mathrm{citrate}$ and (c) with ${\mathrm{Na}}_{3}-\mathrm{citrate}$ and EDTA (mixture) as shown in Figure 4. The ZnS thin film deposited without complexing agents was amorphous, whereas those formed with complexing agents had a broad ZnS peak at 28°. Complexing agents produced ZnS thin films thicker (over 100 nm) and smoother than those without the complexing agent (thickness below 50 nm). Films without complexing agents, with ${\mathrm{Na}}_{3}-\mathrm{citrate}$, and combined EDTA + ${\mathrm{Na}}_{3}-\mathrm{citrate}$ had 85%, 65%, and 70% transmittance and optical band gaps of 3.94 eV, 3.87 eV, and 3.84 eV, respectively. The film with ${\mathrm{Na}}_{3}-\mathrm{citrate}$ and EDTA had 9.12 × 10^10^ carrier concentration and 5.98 cm^2^V mobility.

**Figure 5:**
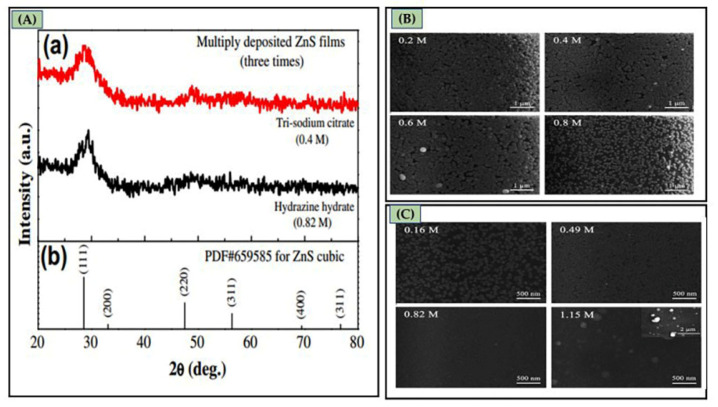
(**A**) XRD analysis of CBD ZnS thin films using varied amounts of (**a**) tri-sodium citrate, and hydrazine hydrate, and (**b**) the ZnS standard value from PDF card: 65-9585. (**B**) SEM images of ZnS thin film with tri-sodium citrate concentration. (**C**) SEM images of ZnS thin film with hydrazine hydrate concentration. Reproduced with permission from *Journal of Alloys and Compounds* **2014**, *588*, 228–234. Copyright © 2014, Elsevier [86].

Liu et al. [86] prepared ZnS on glass substrates using CBD. The impact of several complexing agents (tri-sodium citrate and hydrazine hydrate) and their concentrations on the structure, composition, morphology, optical properties, and growth mechanism of ZnS thin films were examined. XRD study in Figure 5A shows that ZnS thin films (tri-sodium citrate and hydrazine hydrate concentration) have a zinc-blende structure with a pure cubic phase. These 2θ peaks were compared to the ZnS standard value from the PDF card: 65-9585. ZnS thin film formed utilizing 0.8 M tri-sodium citrate had large voids and spherical particles with sizes less than 100 nm and were not very densely packed (see Figure 5B). ZnS films produced with 0.49 M and 0.82 M hydrazine hydrate contain homogenous, densely compacted 20–30 nm small particles (see Figure 5C).

**Figure 6:**
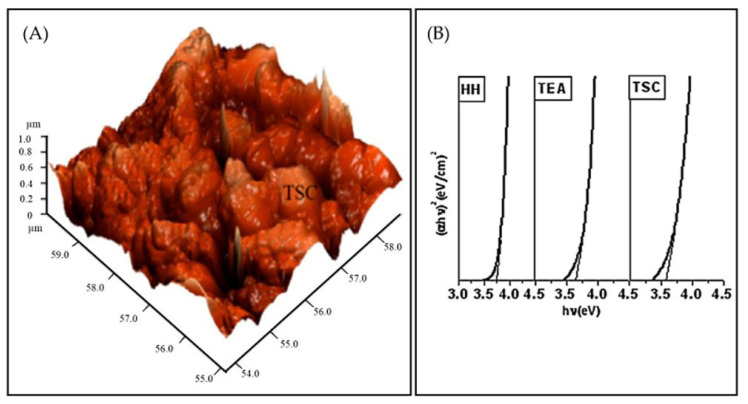
(**A**) 3D AFM measurement with scanning area 5 m × 5 m for ZnS film with TSC complexing agent. (**B**) ${\left(\alpha \mathrm{hv}\right)}^{2}$ versus hv graph for ZnS films with different complexing agents. Reproduced with permission from *Optik* **2014**, *125*, 5727–5732. Copyright © 2014, Elsevier [115].

Regarding the growth mechanism of ZnS in the tri-sodium citrate-ammonia system, ${\left[\mathrm{Zn}{\left(\mathrm{citrate}\right)}_{n}\right]}^{2+}$, ${\left[\mathrm{Zn}{\left({\mathrm{NH}}_{3}\right)}_{3}\right]}^{2+}$, and ${\left[\mathrm{Zn}{\left({\mathrm{NH}}_{3}\right)}_{4}\right]}^{2+}$ were formed with stability constants of 10^8.3^, 10^6.6^, and 10^8.9^, respectively. In the hydrazine-ammonia system, ${\left[\mathrm{Zn}{\left(N_{2}H_{4}\right)}_{3}\right]}^{2+}$, ${\left[\mathrm{Zn}{\left({\mathrm{NH}}_{3}\right)}_{3}\right]}^{2+}$, and ${\left[\mathrm{Zn}{\left({\mathrm{NH}}_{3}\right)}_{4}\right]}^{2+}$ have stability constants of 10^5.5^, 10^6.6^, and 10^8.9^, respectively. If the stability constant is low, complex ions release Zn^2+^ ions more promptly, and ZnS and $\mathrm{Zn}{\left(\mathrm{OH}\right)}_{2}$ colloid particles develop rapidly. Complex ions release Zn^2+^ ions slowly if the stability constant is high, slowing ZnS deposition in solution or on the substrate. ${\left[\mathrm{Zn}{\left(\mathrm{citrate}\right)}_{n}\right]}^{2+}$ in the tri-sodium citrate system is more stable than ${\left[\mathrm{Zn}{\left(N_{2}H_{4}\right)}_{3}\right]}^{2+}$ in the hydrazine hydrate system. The hydrazine hydrate system generated more $\mathrm{Zn}{\left(\mathrm{OH}\right)}_{2}$ colloidal particles, which make the reaction solution milky and murky. $\mathrm{Zn}{\left(\mathrm{OH}\right)}_{2}$ nuclei on the substrate adsorb and hydrolyze thiourea to produce ZnS. The nucleation density of $\mathrm{Zn}{\left(\mathrm{OH}\right)}_{2}$ on the substrate affected the formation of high-quality ZnS thin films [86]. Applying hydrazine hydrate leads to a larger nucleation density of $\mathrm{Zn}{\left(\mathrm{OH}\right)}_{2}$ nuclei on the substrate than tri-sodium citrate; in this scenario, ZnS thin films with small and fine particles formed. Deepa et al. [115] reported the impact of various complexing agents (hydrazine hydrate (HH), tri-sodium citrate (TSC), and tri-ethanolamine (TEA)) on the formation mechanism and characteristics of CBD-synthesized ZnS thin films. All films using a distinct complexing agent were polycrystalline structures with different orientations. This was due to the zinc ions’ rates being released depending on the complex’s stability; the deposition rate would vary with various complexing agents resulting in varied crystallite orientations. Figure 6A shows the AFM for film with TSC. The root means square (R_q_) values of surface roughness values for ZnS films with HH, TEA, and TSC were 71.06 nm, 87.39 nm, and 123.6 nm, respectively. Films prepared using HH were smoother than those produced using TEA and TSC. Film surfaces with TSC were roughest. The transmittance of films using HH, TEA, and TSC was 87%, 60%, and 50%, respectively. The film with HH had the highest transmittance due to its smoother surface and larger grains. HH, TEA, and TSC films had direct band gaps of 3.73 eV, 3.64 eV, and 3.57 eV, respectively (see Figure 6B). ZnS film with TSC had the highest emission peak~(at wavelength: 422.5 nm) followed by TEA (at wavelength: 424.5 nm) and HH (at wavelength: 416.2 nm).

Thus, the literature had proven that complexing agents are crucial to ZnS thin film development and homogeneity in CBD [98,115]. Although ZnS thin films can be produced utilizing the non-toxic complexing agents [86,112,115] described above, the films had a rough shape, a poor growth rate, and a discontinuity microstructure. Hydrazine hydrate produces a smoother film. The high stability constant of zinc complexes in the solution favors gradual zinc ion release, thus diffusion and coalescence are accomplished before second-layer nucleation [115]. It is widely reported that ammonia and hydrazine are the most preferred complexing agents for ZnS films prepared by CBD [105].

#### 3.4.2. Influence of Concentration Ratio of the Reactants

The deposition fluid reactant concentration zinc sulfate heptahydrate and thiourea [Zn]/[S] ratio affected CBD production and the characteristics of ZnS thin films. The origin of the reactants affects the product’s composition, which can influence the deposited thin layer’s physical and chemical properties. By modifying the composition of the reactive solution, the rivalry between homogeneous and heterogeneous nucleation processes can be controlled to promote the formation of thin films [81]. Li et al. [51] investigated the effect of varying the concentrations of the [Zn]/[S] ratio in an aqueous solution with 3:1, 1:1, 1:3, 1:6, and 1:9. In the cases where the [Zn]/[S] ratio was 3:1 or 1:9, no deposition was obtained. [Zn]/[S] ratios ranging from 1:1 and 1:6 were used to form ZnS films. At constant deposition time, the thickness of the film grew as the [Zn]/[S] ratio was raised from 1:1 to 1:6. The optimum [Zn]/[S] film ratio was 1:6 exhibiting pure ZnS films with a wurtzite structure, superior optical transmission (over 85%) in the visible region, and a band gap of 3.65 eV.

Abel et al. [94] investigated the influence of the metallic precursor concentration of zinc acetate dihydrate $\mathrm{Zn}{\left({\mathrm{CH}}_{3}\mathrm{COO}\right)}_{2}.2H_{2}O$ on CBD-ZnS thin films. The following chemical concentrations were determined: 0.1, 0.2, and 0.3 M. As the zinc acetate solution concentration raised from 0.1 M to 0.3 M, the grain size increased from 22.3 to 30.2 nm, and the band gap energy decreased from 3.5 eV to 2.6 eV.

Zhong et al. [58] synthesized ZnS thin films by immobilizing 0.03 M zinc sulfate heptahydrate (${\mathrm{ZnSO}}_{4}.7H_{2}O$) with varying amounts (0.1 to 0.5 M) of thiourea ($\mathrm{SC}{\left({\mathrm{NH}}_{2}\right)}_{2}$) and ammonia (${\mathrm{NH}}_{4}\mathrm{OH}$) (0.3 to 3 M). The order of adding chemicals was crucial for ZnS film development. At $\mathrm{SC}{\left({\mathrm{NH}}_{2}\right)}_{2}$ concentrations 0.2 M and 0.3 M, the film appeared grain by grain; however, at 0.4 M and 0.5 M it formed a continuous layer. This was due to the redundant $\mathrm{SC}{\left({\mathrm{NH}}_{2}\right)}_{2}$’s surplus S^2−^ ions, which create powdery ZnS colloids on the fully grown film. Thickness and deposition rate increased as $\mathrm{SC}{\left({\mathrm{NH}}_{2}\right)}_{2}$ molar concentration increased due to an increased S^2-^ concentration, which accelerated Zn^2+^ and S^2−^ combination in a homogenous process. The visual transmittance for $\mathrm{SC}{\left({\mathrm{NH}}_{2}\right)}_{2}$ concentrations below 0.3 M was about 80%; however, it dropped after 0.4 M. The band gap ranges from 3.73 to 3.79 eV and decreased as $\mathrm{SC}{\left({\mathrm{NH}}_{2}\right)}_{2}$ moles increased.

**Figure 7:**
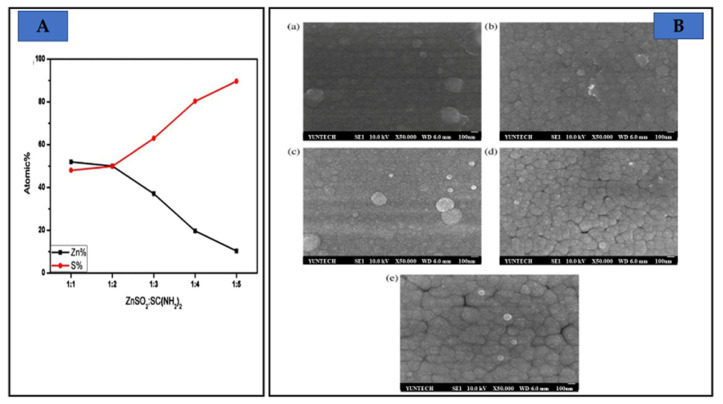
(**A**) Atomic percent variation of Z and S in ZnS thin film. (**B**) SEM images of CBD-ZnS films formed with concentration ratios of ${\mathrm{ZnSO}}_{4}/\mathrm{SC}{\left(NH_{2}\right)}_{2}$ (**a**) 1:1, (**b**) 1:2, (**c**) 1:3, (**d**) 1:4, and (**e**) 1:5. Reproduced with permission from *Applied Surface Science* **2013**, *264*, 213–218. Copyright © 2013, Elsevier [18].

Liu et al. [18] varied $\mathrm{SC}{\left({\mathrm{NH}}_{2}\right)}_{2}$ concentrations (0.03, 0.06, 0.09, 0.12, and 0.15 M) for CBD ZnS. The ratios of ${\mathrm{ZnSO}}_{4}$/$\mathrm{SC}{\left({\mathrm{NH}}_{2}\right)}_{2}$ were 1:1, 1:2, 1:3, 1:4, and 1:5. As seen in Figure 7A, when ${\mathrm{ZnSO}}_{4}$/$\mathrm{SC}{\left({\mathrm{NH}}_{2}\right)}_{2}$ was 1:2, the atomic % of Zn and S was nearly 50% each. Zn atomic % is larger in the film if ${\mathrm{ZnSO}}_{4}$/$\mathrm{SC}{\left({\mathrm{NH}}_{2}\right)}_{2}$ was more than 1:2. If ${\mathrm{ZnSO}}_{4}$/$\mathrm{SC}{\left({\mathrm{NH}}_{2}\right)}_{2}\prime s$ ratio was less than 1:2, and the percentage of atomic Zn was reduced. The ${\mathrm{ZnSO}}_{4}$/$\mathrm{SC}{\left({\mathrm{NH}}_{2}\right)}_{2}$ with concentration ratios of 1:1, 1:2, and 1:3 were smooth and compact; however, films with concentration ratios of 1:4 and 1:5 have surface defects (see Figure 7B). The concentration of ${\mathrm{ZnSO}}_{4}$ was changed from 0.03, through 0.05, 0.07, and 0.09 M, to 0.11 M. The energy gap lowered from 3.980 to 3.885 eV as the ${\mathrm{ZnSO}}_{4}$ concentration increased from 0.03 to 0.11 M.

Christinal et al. [109] investigated the effect of sulfur concentrations on the growth of CBD-ZnS thin films. The concentration of thiourea varied between 0.4 M and 0.6 M. The most prominent XRD peaks occurred at the (1 1 1) zinc blende structure. FESEM analysis showed the ZnS particle size increased as the sulfur concentration increased. The UV analysis revealed an optical transmittance greater than 70% and a band gap energy between 3.50 and 3.75 eV. Wei et al. [85] studied the effect of thiourea (0.15 M, 0.45 M, 0.75 M, and 1.05 M) and ammonia (0.1 M, 0.4 M, 0.7 M, and 1.2 M) concentrations on ZnS thin films while preserving other reagent concentrations. According to [85], thiourea $(\mathrm{SC}\left({\mathrm{NH}}_{2}{)}_{2}\right)$ concentration did not affect ZnS thin film surface morphology. However, ammonia concentration affects film morphology. Thin films produced with 1.2 M ammonia had cracks, but those deposited with 0.7 M ammonia were denser and smoother. Those results could be explained in terms of ZnS deposition kinetics and reaction mechanism. The ${\left[\mathrm{Zn}{\left(N_{2}H_{4}\right)}_{3}\right]}^{2+}$ ions predominate at 0.1 M ammonia. On the substrate, colloidal particles formed through a homogeneous reaction in solution progressively adhered. ZnS film growth was cluster-by-cluster. ${\left[\mathrm{Zn}{\left({\mathrm{NH}}_{3}\right)}_{3}\right]}^{2+}$ ions are significant for deposition at 0.7 and 1.2 M ammonia, in which ZnS films grown on a substrate ion-by-ion are denser and smoother. The transmittance was greater than 70% between 350 and 900 nm, and the optical band gap of ZnS thin films varied between 3.76 and 3.78 eV. These results demonstrated that a high-quality ZnS thin film requires a specific ratio of reactant concentrations.

#### 3.4.3. Influence of Stirring Speed

**Figure 8:**
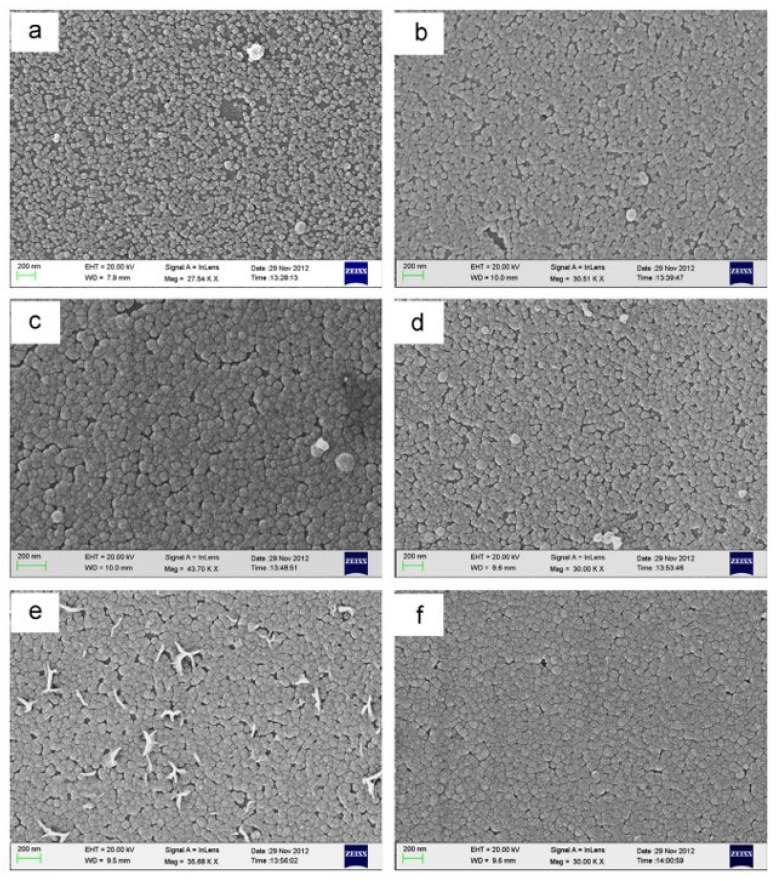
FESEM images of samples deposited at 50 °C (**a**,**b**), 70 °C (**c**,**d**), and 90 °C for (**e**,**f**). The films deposited for2 h (**a**,**c**,**e**), and 2.5 h (**b**,**d**,**f**). Reproduced with permission from *Materials Science in Semiconductor Processing* **2014**, *18*, 28–35. Copyright © 2014, Elsevier [106].

Typically, the CBD process stirred the ZnS solution to ensure the chemical components are uniformly distributed [106,117]. This also inhibits colloidal particles and grain size, disrupting particle diffusion to the surface and absorbing particles on the substrate [43]. However, Dona and Herrero [80] found that ZnS film growth was independent of CBD solution stirring. Ke et al. [106] reported that the static reaction bath (unstirred condition) was beneficial for the creation of heterogeneous ZnS thin films and improved the structural and optical features compared to prior literature and stirring conditions. Figure 8 displayed the ZnS thin films deposited for 2 and 2.5 h. Throughout the deposition, the temperature reaction solutions were maintained at 50 °C, 70 °C, and 90 °C. Ke et al. [106] found that ZnS thin films deposited at 90 °C without siring showed improved morphology. ZnS films deposited over 2 h without stirring had better crystallinity and all films had band gap energy ranges between 3.93 and 4.06 eV.

Hubert et al. [118] concurred that the stirring rate did not affect ZnS thin film growth during deposition times lowers than 30 min. However, after 30 min of deposition, stirring affects ZnS thin film formation. It showed contradictory results between the references [80,106]. Zhang et al. [117] observed that stirring speed improves cluster adsorption on ZnS films. Zhang et al. [117] discovered that stirring speeds had an effect on the thickness of the film but did not affect its crystallization and optical properties. The films showed similar optical properties, independent of their stirring speeds. The films exhibited high transmittance (70–88%) in the visible spectrum, and the band gap of ZnS was around 3.63 eV. Onal and Altiokka [119] demonstrated that ZnS films obtained without stirring the solution exhibited a low XRD peak intensity, low thickness, and lower band gap values than films obtained by stirring the solution. The resulting films with the stirring solution were denser and less transparent. Typically, metal ions are heavier than water molecules. When the solution was stirred and mixed, centrifugal force discharged metal ions into the bath container and the surface of the glass substrate.

The literature discussing the effect of stirring on the ZnS thin film contradicts the results. Generally, stirring is advantageous because it forces particles to interact throughout the process and introduces solvent components into interaction with the solute [81]. With stirring, the solution obtained was more transparent [85]. As the rate of stirring increased, Onal and Altiokka [119] demonstrated that the ZnS thin film efficiently adhered to the glass substrate. Several references were continuously stirred at varying rates of stirring speed for preparing ZnS thin films using CBD [58,76,85,105].

#### 3.4.4. Influence of Humidity

**Figure 9:**
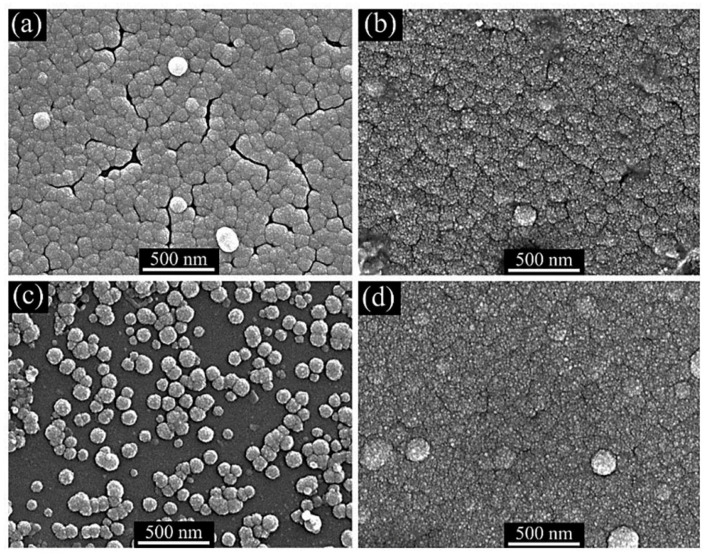
SEM images of ZnS films deposited under a relative humidity of (**a**) 60%, (**b**) 70%, (**c**) 80%, and (**d**) hermetic system. Reproduced with permission from *Applied Surface Science* **2014**, *307*, 724–730. Copyright © 2014, Elsevier [69].

In operational use, the CBD process can be carried out either in a closed reaction container (hermetic CBD system) [51,80,85] or an open reaction container (open CBD system) [114,120]. Hermetic CBD prevents evaporation and environmental interference by closing the chemical bath and substrate [85]. The volume of the system is constrained to achieve thermal equilibrium, and the bath–substrate interface is scarcely disturbed [43]. In an open CBD system, ambient humidity interferes with the gas–liquid contact. To date, no systematic research has been conducted on the influence of humidity on CBD-ZnS thin films. Lin et al. [69] examined the influence of humidity on CBD-ZnS thin film synthesis. The experimental conditions were done with hermetic CBD and open CBD under a relative humidity of 60%, 70%, and 80%. For XRD structures, all films had an amorphous form for both open CBD and hermetic CBD. Film morphology was highly sensitive to relative humidity (see Figure 9a–c). Hermetic CBD produced a compact conformal thin layer with tightly packed microstructures (see Figure 9d). Open CBD films had many cracks and powdery samples. The X-ray photoelectron spectrum reveals that relative humidity increases the concentration of ZnO compounds in an open system. The hermetic CBD film had the highest average visual transmittance (77%) in the wavelength range of 400–800 nm, exceeding all other films. The hermetic CBD-ZnS film exhibited improved transmission and morphology rather to the open CBD films. Regarding Shobana et al. [42], a closed and isolated apparatus (hermetic CBD) is preferable for producing a thin film free of impurities.

#### 3.4.5. Influence of Deposition Temperature

Bath temperature can affect the chemical reaction rate [81]. As temperature rises, the complex dissociates causing an increase in the kinetic energy of the particles, which increases ion interaction and deposition at substrate volume nucleation centers. Generally, CBD methods precipitated a chemical from a solution onto a substrate at 303–353 K [69].

Zhou et al. [101] prepared CBD ZnS thin films at different deposition temperatures (75 °C, 80 °C, 85 °C, 90 °C, and 95 °C) to study their properties. The films were annealed at 200 °C. As deposition temperature increased, ZnS thin films thickened, and average particle size increased. Thickness was in the range of 73–200 nm. Above 600 nm, the optical transmittance of the films exceeded 75%. As the deposition temperature increased, transmittance dropped. The film with low deposition temperature is thinner and more transparent. This might be attributed to the varied thicknesses and roughness. Gode et al. [54] prepared the films at deposition temperatures of 60 °C, 70 °C, and 80 °C. Deposition temperatures of 60 °C and 70 °C produced amorphous films. The best diffraction peak was observed at deposition temperatures of 80 °C for deposition times of 4.5 h. Liu et al. [18] varied the bath deposition temperatures for ZnS thin films through 75 °C, 80 °C, and 85 °C. Deposition temperatures enhanced film thickness. Dona and Herrero [80] found that with the deposition temperature elevated from 70 to 80 °C, CBD-ZnS thin films thickened. Jawad and Alioy [121] used the CBD approach to increase Cd_1-x_Zn_x_S film thickness from 60 °C to 80 °C. This was because the hydrolysis of thiourea increased as the temperature increased.

#### 3.4.6. Influence of Deposition Time

Deposition time is a significant factor affecting the crystal structure and optical characteristics of ZnS films deposited using CBD [43,54]. Liu et al. [100] investigated the varied deposition times (20, 40, 60, and 80 min) on the properties of CBS-ZnS. The surface of a film formed at 80 °C was not smooth and dense even after the deposition times exceeded 60 min. The average transmittance decreased as deposition time increased. This was because the average transmittance dropped as the film’s thickness increased. For example, at a constant bath temperature (80 °C), the transmittance (85.2%, 80.3%, 76.8%, and 74.4%) and thickness raised (98 nm, 135 nm, 177 nm, and 273 nm) as deposition time increased (20 min, 40 min, 60 min, and 80 min). Luque et al. [91] studied the different deposition times of 30, 60, and 90 min for ZnS thin film performance. The ZnS growth on glass was more homogenous for the material with 90 min of reaction, exhibiting strong transmission (80% in the spectral region of 300 to 800 nm), electrical resistivity (10^6^ Ω cm), and an optical band gap of 3.62 eV.

Kumar et al. [89] reported varied deposition times of 60, 80, 100, and 120 for CBD-prepared ZnS. XRD revealed that time deposition films increase crystallinity and crystallite size. The particle size increased (193–242 nm), thickness increased (239–590 nm), the transmission of the films was over 70%, and band gap values declined (3.68 eV–3.68 eV) as deposition time increased. XPS studies showed that ZnS thin film formed at 120 min had two peaks centered at 1044.9 eV and 1021.8 eV, matched to the Zn2p_1/2_ and Zn2p_3/2_. The peaks related to the Zn^2+^. The evaluation of electrical performance parameters revealed an enhanced ideality factor in heterostructures manufactured with a deposition time of 60 min compared to other films.

Gode et al. [54] demonstrated that deposition time significantly influences ZnS thin film crystallite, grain size, thickness, and optical properties. They showed the impact of longer deposition time on the growth of ZnS films using CBD. When the deposition temperature was obtained at 80 °C, the glass substrates were then put into the solution. To evaluate the rate of growth, the deposition times were monitored for 3, 3.5, 4, and 4.5 h. The ZnS (0 0 8) peak grew narrower as deposition time increased, indicating improved crystallinity. As deposition time increased, thin film grain size (40–82 nm) and thickness (403–934 nm) increased. The film’s transmittance in the visible region was 66–87%. The energy dispersive X-ray analysis (EDX) revealed zinc and sulfur layers in the films. The average atomic ratio of S/Zn was computed to be 0.51, 0.56, 0.57, and 0.58 for deposition durations of 3, 3.5, 4, and 4.5 h, respectively. All films had metal-rich surfaces with ratios below the stoichiometric ratio (S/Zn = 1). A deviation in S/Zn ratios might be attributable to the greater oxygen atomic percentage. Oxygen might occur from the atmosphere or the bath solution. For the layer deposited at 4.5 h, the electrical conductivity is 4.67 × 10^−10^ Ωcm and the resistivity is 2.14 × 10^9^ Ωcm.

Haddad et al. [122] demonstrated that deposition time influences the structural, morphological, transmission, and photoluminescent properties of CBD-ZnS thin films. Without stirring, deposition times varied from 2 to 6 h. Increasing deposition time increases XRD peak intensity, indicating improved film crystal quality. In their situation, after a few minutes of deposition, ZnO nuclei surrounded by S^2−^ ions arise. With longer deposition times, greater S^2−^ ions surround ZnO nuclei (poor Zn–O interaction), and O^2-^ ions diffused from the inner surface leading to ZnS via sulfidation. Deposition time raises crystallite size from 2.6 to 10 nm and decreases the lattice parameter. With an increase in deposition, ZnS nucleation stops, and ZnS film growth was related to an increase in average particle size rather than continuous primary particle nucleation and deposition. Film thickness increased from 300 to 610 nm for 2 to 3 h and then lowered to 530 nm for 4, 5, and 6 h. The film’s transmittance was independent of deposition time but absorption was dependent. Visible spectrum transmittance ranged from 60 to 83% from 400 to 800 nm. Photoluminesce data showed deposition time affects ZnS thin film emissions. The 5 h film had the most UV–blue emission, whereas the 4 h film had the lowest.

Goudarzi et al. [47] reported that CBD ZnS film formation began approximately 15 min after reactant mixing and lasted approximately 7 h. At wavelengths exceeding 350 nm, ZnS film with 0.5 h deposition (18 nm of thickness) transmitted more than 85%. The 100 nm-thick layers transmitted greater than 70% of visible light. Lower deposition times result in improved transmittance at shorter wavelengths, which increased short-circuit current. Thus, the literature revealed that the deposition time is a critical factor that affects the growth, structural, and optical properties of CBD ZnS development. Optimizing buffer layer thickness is required for ZnS to improve solar cell performance [47].

#### 3.4.7. Influence of pH Value

**Figure 10:**
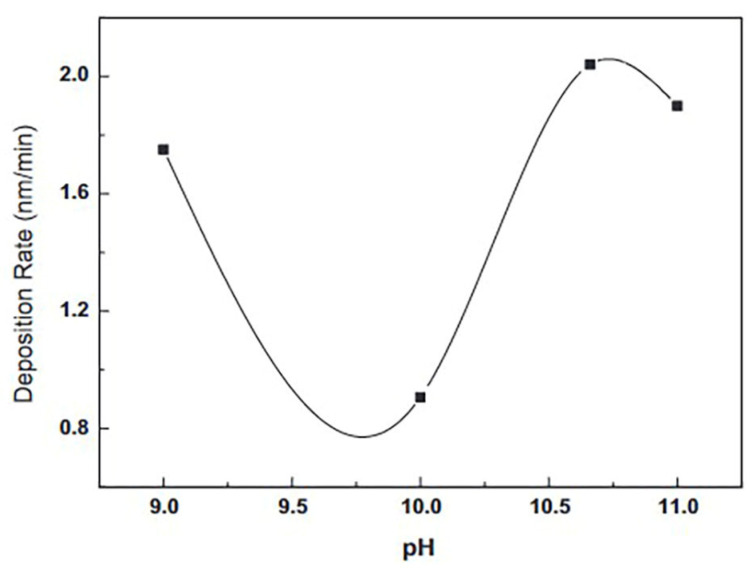
The deposition rate of ZnS thin films using CBD. Reproduced with permission from *Materials Science in Semiconductor Processing* **2013**, *16***,** 1753–1758. Copyright © 2013, Elsevier [105].

Reaction rate and deposition rate rely on supersaturation and rate of MX formation (where M and X are metals and O^−^/OH^−^ ions, respectively) [81]. The deposition rate is proportional to the ratio of film thickness to deposition time [105]. To produce high-quality thin films, precursor solutions must contain hydroxy ions (OH^−^) [81]. The production of a thin film relies on reaction mixture pH, and pH is determined by OH^-^ ions. pH affects the equilibrium between complexing agents and water [81]. Lekiket and Aida [105] reported that reaction fluid pH affected CBD-ZnS thin films. They discovered that the rate of deposition was pH-dependent. The results varied from 0.9 to 2 nm/s for pH levels between 9 and 11. As seen in Figure 10, the film formed at a pH of 10 had a reduced deposition rate due to the increased ZnS solubility at this pH. In thermodynamic research, Hubert et al. [123] computed ZnS solubility in an ammonia solution reporting a maximum solubility at pH = 10. At pH values ranging from 9 to 10.66, an XRD peak indicated that the films were crystalline. The average transmittance stands between 75% and 80%, while the band gap energy ranges from 4.0 eV to 4.2 eV for all pH values. Selma and Alioy [121] reported that the optimal ${\mathrm{NH}}_{3}$ concentration required to bind Zn^2+^ to their compound in the bath deposition is pH = 10. Nasr et al. [39] studied CBD zinc sulfide thin films at pH 10 and 11.5. At pH = 10, they obtained a ZnS coating with high crystallinity with transmission ranging from 20 to 46%. However, for the ZnS film at pH =11.5, transmission is at a maximum between 55 and 71%, but there is no discernible XRD diffraction peak. Both pH = 10 and pH = 11.5 resulted in a band gap of 3.78 eV for the ZnS film. Kang et al. [114] synthesized CBD ZnS thin films in an acidic solution with a pH range of 5.0 to 6.5. They utilized various pH levels of ethylenediamine tetra acetic acid disodium salt (Na_2_EDTA). All films had a nanocrystalline structure. The direct band gap (3.78 to 3.91 eV) varies with solution pH. These show the films from the same material formed in acidic and alkaline environments may exhibit distinct characteristics.

**Figure 11:**
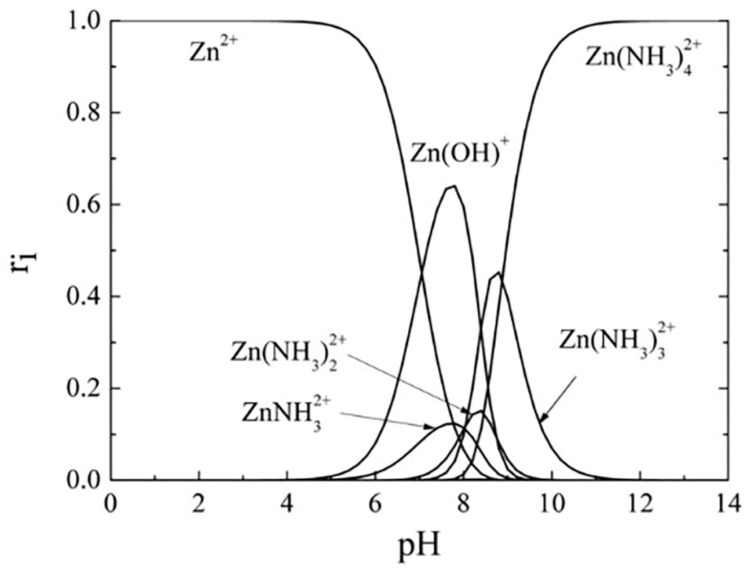
CBD composition of Zn-OH-${\mathrm{NH}}_{3}\text{-}H_{2}O$. Reproduced with permission from *Materials Chemistry and Physics* **2012**, *136*, 386–393. Copyright © 2012, Elsevier [76].

Tec-Yam et al. [76] reported the CBD-ZnS composed of Zn-OH-NH_3_-$H_{2}$O components at different pH ranges. Figure 11 shows Zn^2+^ ions and no criteria for ZnS film production at pH 5 (acidic solution). pH ranging from 6 to 10 produced undesired intermediate complexes, precipitates, and no ZnS was observed. When the pH exceeds 10, ${\left(\mathrm{Zn}{\left(\mathrm{NH}\right)}_{3}\right)}_{4}^{2+}$ is more likely to occur. ${\left(\mathrm{Zn}{\left(\mathrm{NH}\right)}_{3}\right)}_{4}^{2+}$ and ${\left(\mathrm{Zn}{\left(\mathrm{NH}\right)}_{3}\right)}_{3}^{2+}$ have stability constants of 10^8.9^ and 10^6.6^, respectively. Higher stability constants slow Zn^2+^ discharge from complexes [11]. In line with the literature [11,39,76,85], ${\left(\mathrm{Zn}{\left(\mathrm{NH}\right)}_{3}\right)}_{4}^{2+}$ is the most preferred ion for ZnS production. The CBD-ZnS thin film is generated as follows in the presence of ${\mathrm{NH}}_{3}$ as a complexing agent in the CBD-ZnS alkaline solution: ${\left[\mathrm{Zn}{\left({\mathrm{NH}}_{3}\right)}_{N}\right]}^{2+}🡲{\;\mathrm{Zn}}^{2+}+{\;\mathrm{nNH}}_{3},\;\mathrm{SC}{\left({\mathrm{NH}}_{2}\right)}_{2}$+$2{\mathrm{OH}}^{-}🡲{\;S}^{2-}+{\;\mathrm{CN}}_{2}H_{2}+2H_{2}O,{\;\mathrm{Zn}}^{2+}+{\;S}^{2-}🡲\;\mathrm{ZnS}$ [11]. The ideal pH range for producing high-quality CBD-ZnS films was 10 to 12. These ZnS films were deposited at the optimal pH by varying alkaline complexing agents. Through the introduction of ${\mathrm{NH}}_{3}$, a complexing agent, the ${\left(\mathrm{Zn}{\left(\mathrm{NH}\right)}_{3}\right)}_{4}^{2+}$ zinc complex was formed, hence Zn^2+^ ions slowly released. Reduced deposition due $\mathrm{to}\;{\left(\mathrm{Zn}{\left(\mathrm{NH}\right)}_{3}\right)}_{4}^{2+}$ can improve ZnS film quality.

In recent years, a few researchers [98,124,125,126] had developed and employed species distribution diagrams (SDDs) and solubility curves (SCs) to undertake physicochemical analysis to analyze the synthesis of ZnS through the distinct species produced in the chemical solution as a function of the pH. It may provide the researcher with ideas before developing ZnS thin films.

#### 3.4.8. Influence of Precursor Type

Multiple investigations have confirmed that the chemical effects, such as Zn precursor, influence ZnS thin film growth and physical properties. Hong et al. [11] examined that zinc sulfate (${\mathrm{ZnSO}}_{4}$), zinc acetate $\left(\mathrm{Zn}{\left({\mathrm{CH}}_{3}\mathrm{COO}\right)}_{2}\right)$, and zinc chloride (${\mathrm{ZnCl}}_{2}$) influenced CBD ZnS thin film growth rate, structure, and optical characteristics. Utilizing ${\mathrm{ZnSO}}_{4}$, $\mathrm{Zn}{\left({\mathrm{CH}}_{3}\mathrm{COO}\right)}_{2}$, and ${\mathrm{ZnCl}}_{2}$, film thickness was measured to be ∼90 nm, ∼60 nm, and ∼30 nm, respectively. The influence of film thickness on Zn sources was correlated with complex ion stability constants, in which ${\mathrm{ZnSO}}_{4}$, $\mathrm{Zn}{\left({\mathrm{CH}}_{3}\mathrm{COO}\right)}_{2}$, and ${\mathrm{ZnCl}}_{2}$ precursors had stability constants of 0.70, 0.78, and 1.5, respectively. Due to slow Zn^2+^ release from ${\mathrm{ZnSO}}_{4}$ with a lower stability constant, a thicker ZnS layer was formed. The XPS results imply that the binding energies of Zn 2p_3/2_ in ZnS films differ significantly based on the Zn precursors, which results in the discrepancy between Zn, S, and O constituents. By utilizing ${\mathrm{ZnSO}}_{4}$, $\mathrm{Zn}{\left({\mathrm{CH}}_{3}\mathrm{COO}\right)}_{2}$, and ZnCl_2_, the E_g_ values were 3.40, 3.49, and 3.44 eV, respectively. Although ZnS thin films from different precursors have comparable E_g_ values, the oxygen content in ZnS can influence E_g_. Khatri and Patel [71] explored the influence of zinc precursors (${\mathrm{ZnCl}}_{2}$, $\mathrm{Zn}{\left({\mathrm{CH}}_{3}\mathrm{COO}\right)}_{2}$, and ${\mathrm{ZnSO}}_{4}$) on ZnS thin growth using CBD. The XRD analysis revealed a hexagonal phase for all zinc precursors. The particle size decreased (27 nm, 25 nm, and 22 nm), thickness increased (37 nm, 39 nm, and 41 nm), the transmittance at wavelength ˃ 350 nm decreased (29%, 25%, and 10%), and band gap increased (4.10 eV, 4.17 eV, and 4.25 eV) for thin films with various zinc precursors ${\mathrm{ZnCl}}_{2}$, $\mathrm{Zn}{\left({\mathrm{CH}}_{3}\mathrm{COO}\right)}_{2}$, and ${\mathrm{ZnSO}}_{4}$, respectively. ZnS thin film thickness was affected by growth rate and zinc precursors in solution during the growth process. All ZnS films have a 348 cm^−1^ first-order Raman shift. The Hall-effect measurement showed carrier concentration (10^15^ to 10^17^/cm^3^), indicating the n-type ZnS thin film. ZnS thin films produced with ${\mathrm{ZnCl}}_{2}$ have the highest carrier mobility. Both studies [11,71] imply zinc precursors affect CBD-ZnS physical and growth qualities.

CBD-ZnS films comprise ZnS, $\mathrm{Zn}{\left(\mathrm{OH}\right)}_{2}$, and ZnO, hence the identification of ZnS(O,OH) [74]. Ernits et al. [74] evaluated the influence of anion Zn sources on chemically deposited ZnS(O,OH) films. Different Zn precursor salts were used: $\mathrm{Zn}{\left({\mathrm{CH}}_{3}\mathrm{COO}\right)}_{2}.2H_{2}O$, $\mathrm{Zn}{\left(\mathrm{Ac}\right)}_{2}$, ${\mathrm{ZnCl}}_{2}$, ${\mathrm{ZnI}}_{2}$, $\mathrm{Zn}{\left({\mathrm{NO}}_{3}\right)}_{2.}4H_{2}O$ and ${\mathrm{ZnSO}}_{4}.7H_{2}O$. According to their findings, the amount of ZnS in ZnS(O,OH) film depends on the zinc precursor complex’s instability constant. The zinc supply affects the ZnS(O,OH) clusters size and the film thickness. XPS measurements detected contaminants in ZnS(O,OH) films from the sources: $\mathrm{Zn}{\left({\mathrm{NO}}_{3}\right)}_{2}$, ${\mathrm{ZnI}}_{2}$, and $\mathrm{Zn}{\left(\mathrm{Ac}\right)}_{2}$. ZnS(O,OH) deposited from a $\mathrm{Zn}{\left({\mathrm{NO}}_{3}\right)}_{2}$ solution had the widest band gap (3.8 eV) and maximal transmission (˃95%). ZnS(O,OH) buffer layers formed from $\mathrm{Zn}{\left(\mathrm{Ac}\right)}_{2}$ had the highest current densities and efficiency. Both results [11,71] indicated zinc precursors influence the physical and growth properties of CBD-ZnS, while Ernits et al. [74] demonstrated zinc precursors also affect the physical, structural, and efficiency of ZnS(O,OH) buffer layers. According to Sinha et al. [43] and current updated research (Table 3), ${\mathrm{ZnSO}}_{4}$ was discovered to serve as a preferable precursor material for producing ZnS films by CBD.

#### 3.4.9. Influence of Annealing Temperature and Environment

All previously described parameters (Section 3.4.1, Section 3.4.2, Section 3.4.3, Section 3.4.4, Section 3.4.5, Section 3.4.6, Section 3.4.7 and Section 3.4.8) were evaluated before or during the deposition process to determine whether they contributed to the improvement of film growth. After utilizing CBD to deposit on the glass, annealing will be applied. Annealing improves pre-deposited film crystallinity, shape, and optical characteristics (transmissivity) [76,127]. Different films require heat treatment with varying temperatures (200–550 °C) and steps. Thermal energy removes disturbance surface particles during air annealing, hence smoothing the films [43].

Zhou et al. [127] investigated the CBD method’s annealing effect on ZnS thin film prepared on SLG. The ZnS films were prepared without annealing and annealing in the atmosphere for 1 h at 200 °C and 300 °C, respectively. They discovered that annealing temperature affects the morphology and optical properties but not the crystalline structure. The films were amorphous. Before annealing, the film’s primary components were ZnS and ZnO. Without annealing, a certain quantity of $\mathrm{Zn}{\left(\mathrm{OH}\right)}_{2}$ might produce in the ZnS films, and after annealing $\mathrm{Zn}{\left(\mathrm{OH}\right)}_{2}$ degraded ZnO. For pre-annealing ZnS films, the surface area was tiny, not uniform, and not fined. After annealing, the surface became dense and flat, and the particles dispersed. The ZnS film’s transmissivity exceeded 80%, whereas the films without annealing had higher transmissivity than at 200 °C and 300 °C for 1 h.

**Figure 12:**
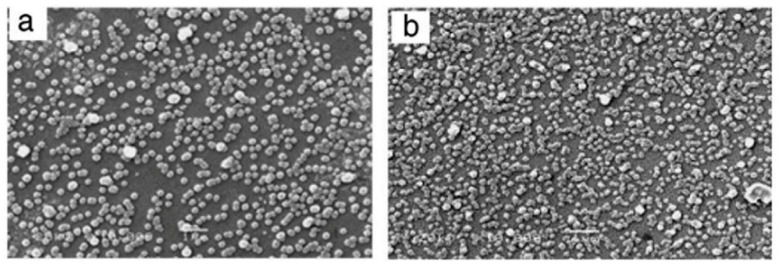
SEM images of (**a**) deposited and (**b**) annealed CBD-ZnS thin films. Reproduced with permission from *Surface & Coatings Technology* **2013**, *228*, 146–149. Copyright © 2013, Elsevier [101].

Research [101,128,129] discovered that the effect on ZnS films resulted in a change in optical properties but had no effect on the crystalline structure (either amorphous or crystalline film on film deposition). Oliva et al. [128] synthesized CBD-ZnS films by annealing the films at 200 °C and 400 °C. According to their findings, the crystal structure of the as-grown and annealed samples did not change. XRD confirms ZnS films had a cubic and sphalerite-type structure. The band gap energy of the films was 3.70 eV and 3.45 eV for as-grown and annealed films, respectively. Both the as-grown and post-annealing transmissivities of the films were observed to be between 80% and 70%. The results of Oliva et al. [128] found that annealing ZnS films lowered optical band gap energy and transmittance without affecting the crystal structure. Zhou et al. [101] examined CBD ZnS thin films before and after annealing in air and discovered that annealing affects the morphology and optical properties but has no effect on the film’s crystallinity. All deposited and annealed films at 200 °C were amorphous. The annealed ZnS thin film had a more homogeneous, dense surface morphology and particle distribution than the deposited films (see Figure 12). ZnS oxidised to ZnO and grew smaller particles during air-annealing. The annealed ZnS film had poorer transmittance than the deposited in the 350–800 nm wavelength range. Annealing effect uniforms the ZnS thin film surface, reduces defect density and lowers transmittance. Khalil et al. [129] investigated CBD ZnS thin film post-annealing at 100–400 °C in air during 1 h. All deposited and annealed films exhibited crystalline structures. There was no impurity discovered other than ZnS, which matched the hexagonal structure of wurtzite. After annealing, the ZnS crystal structure expanded and became aggregated. The thickness of the films increases (from 160.13 to 203.3 nm) as the post-annealing temperature rises. Both transmittance and E_g_ decreased as a result of increased post-annealing, greater particle size, and morphological modification (in the range of 3.9653 eV to 3.6888 eV).

**Figure 13:**
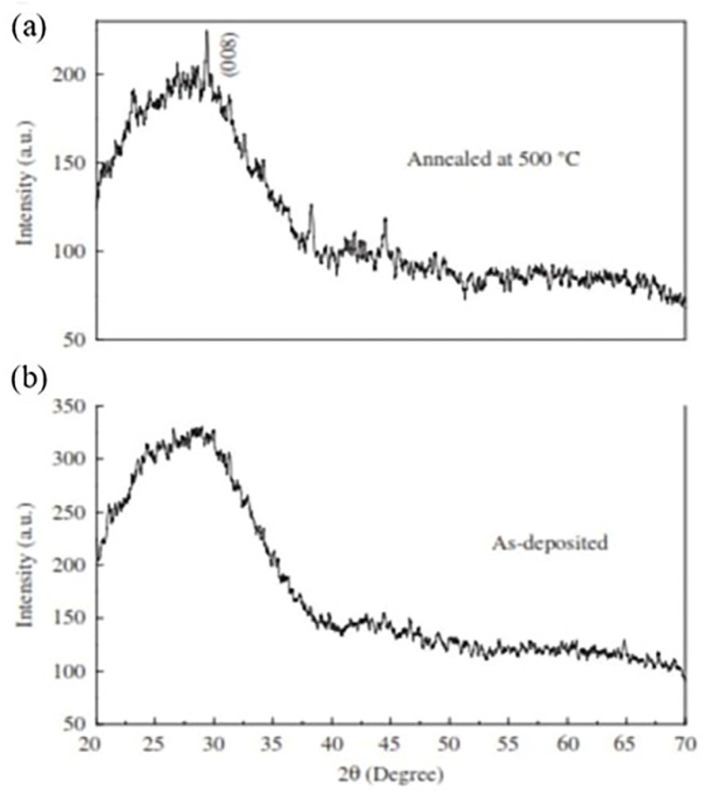
X-ray diffraction (005-0566 JCPDS reference) data of ZnS films (**a**) as-deposited and (**b**) after annealing at 500 °C. Reproduced with permission from *Physica B: Condensed Matter* **2011**, *406*, 1653–1659. Copyright © 2011, Elsevier [130].

According to certain research [97,102,130], the CBD technique yields either amorphous or weakly crystalline films. High-temperature annealing is necessary to increase the film’s crystallinity. Sayed et al. [97] presented a study on the effects of different annealing temperatures on CBD-ZnS films. The films were annealed at temperatures ranging from 150 to 300 °C. XRD results showed the films had dominating intensity diffraction at the (111) plane indicating the preferred orientation of crystallization. The transmittance (77.32–79.43%) and band gap values (3.34–3.45 eV) of the films increased with increasing annealing temperatures. Gode [130] reported that the films were annealed at 100 °C to 500 °C in 100 °C increments in the air for one hour. The study found that the films’ optical and crystallization properties depend greatly on the annealing temperature. XRD data demonstrated that the film phase was amorphously deposited initially (see Figure 13a), and heat treatment slightly changed the film’s structure (see Figure 13b). The film showed polycrystalline after 500 °C annealing, which proved that post-deposition annealing boosts crystallinity. Raman spectra of annealed ZnS samples revealed the compositions of first-, second-, and third-order Raman phonons. The direct band gap dropped from 4.01 to 3.74 eV as the annealing temperature increased. Ahn and Um [102] investigated the influence of varying the annealing temperature from 100 °C to 300 °C on the crystallization and optical properties of the ZnS thin deposited on SLG. The ZnS thin film annealed at 100 °C exhibited an amorphous structure, but as the annealing temperature increased (from 200 to 300 °C), the thin film’s crystalline quality enhanced. The average grain size ranged from 134.1 to 178.5 nanometers. Annealing improved ZnS thin film quality by increasing the grain size and the surface became compact and homogeneous as revealed by FESEM images. XPS spectra of the 300 °C-annealed film showed the characteristic Zn-S bond peak. As the annealing temperature increased (100 °C, 200 °C, and 300 °C) the direct energy gap (3.89 eV, 3.85 eV, and 3.82 eV) and surface roughness (28.49 nm, 22.45 nm, and 19.91 nm) decreased. This reduction in the direct energy gap is a result of the increased crystallite size, which decreased the dislocation density.

Despite air atmosphere being described in other references, annealing ZnS films with a different atmosphere had also been mentioned by Shin et al. [52], Cao et al. [90], and Shan et al. [95]. Shin et al. [52] investigated the different annealing temperatures and atmosphere effects on CBD-prepared ZnS thin films on ITO-coated glass substrates. ZnS thin films were annealed at 300–550 °C in a vacuum, $N_{2}$, and $N_{2}$ (95%) + $H_{2}$S (5%). As-deposited ZnS thin film demonstrated amorphous characteristics and had Zn–OH and Zn–S bonds. Annealed thin films had only Zn–S bonds. It showed annealing ZnS thin films eliminates ZnOH phases. The films annealed at various temperatures in a vacuum and N_2_ atmospheres were amorphous or weakly crystalline. The best environment for generating ZnS thin films with high crystallinity was discovered to be $N_{2}$ + $H_{2}$S. S in the $N_{2}$ + $H_{2}$S atmosphere boosted the annealed ZnS thin film’s X-ray peak intensity. As-deposited and annealed ZnS thin films in a vacuum, $N_{2}$, and $N_{2}$ + $H_{2}$S have Zn atomic ratios of 50.14%, 49.85%, 46.85%, and 42.02%, respectively. Due to thermal energy-evaporated Zn atoms and H_2_S gas, ZnS thin films annealed in different atmospheres had increased S concentrations. ZnS thin films annealed in vacuum and N_2_ had smaller grain sizes than those annealed in $N_{2}$ + $H_{2}$S. Films annealed at various temperatures and environments had poorer transmittance than the as-deposited film. All the films exhibited moderately visible transmittance (>70%). Depending on the annealing conditions, the direct band gap of the annealed films ranged between 3.89 and 3.5 eV. Cao et al. [90] reported the CBD-ZnS film annealed at 500 °C in Ar/$H_{2}$S. (5%) for one hour. XRD results demonstrate the low peak at 28.5° corresponds to reflections from (1 1 1) planes of cubic structured ZnS and indicates the deposited ZnS thin films showed low crystal quality. After annealing, intense and sharp reflection peaks appeared at 28.5° and 47.5° and 56.3° related with (2 2 0) and (3 1 1) planes of cubic structured ZnS. XRD results demonstrate that annealing ZnS thin films enhances crystallization. Since the majority of ZnO or $\mathrm{Zn}{\left(\mathrm{OH}\right)}_{2}$ was being converted into ZnS, all annealed samples contained an oversupply of S. Shan et al. [95] reported on CBD-ZnS films annealed in a sulfur environment at 500 °C for one hour. The as-deposited ZnS thin films and S powder (0.4, 0.8, and 1.2 mg) were enclosed in a vacuum glass tube for sulfurization with S vapor pressures of 2.0, 4.0, and 6.0 × 10^3^ Pa. Sulfurization pressure affects film crystallographic, morphological, and optical properties. XRD data showed that post-annealing in a sulfur environment helped the anomalous ZnS precursor film become crystalline cubic ZnS and increased crystallinity. The transmission spectra demonstrated that the transmittance (50–80%) of the film rapidly increased when the sulfur pressure rise, suggesting that this will reduce the band gap of the ZnS film. In summary, according to the literature, annealing influenced the morphology and optical properties in particular transmissions and E_g_ of CBD-prepared ZnS films [101,102,127]. In terms of morphology, annealing ZnS thin films cause the growth of particles and smoothing of the surface [101]. High-temperature annealing is necessary to enhance the film’s crystallinity [105,130]. This is because the CBD method on deposited film typically produces amorphous or weakly crystalline films. The ZnS thin film that was deposited formed a combination of ZnS and $\mathrm{Zn}{\left(\mathrm{OH}\right)}_{2}$ phases [131]. Thermal energy increases the crystallinity of the annealed films [52,90]. The film surface becomes defect-free as contaminants and artifacts disappear during annealing, increasing the XRD peak intensity [43]. Annealing lowers optical transmittance by reducing particle space and making particles more compact [101,102]. The observed transmittances for ZnS thin films were 80% and 70% with an annealing temperature [102,127,128]. ZnS with these transmittance levels is appropriate to be utilized as buffer layers in CIGS solar cells replacing CdS films [102].

Thus, numerous parameters from CBD influence the shape, structure, and optical properties of ZnS films. Additionally, other phases during film formation affect CBD-ZnS-deposited thin film properties. Due to this, several publications have observed that the crystallization, structure, morphology, optical properties, and stoichiometry vary depending on the chemical reagents and conditions used in the production of CBD. All the CBD parameters, when combined, can cause several effects on the films, which are sometimes difficult to regulate and recognize, resulting in inconsistent findings across researchers. Due to the repeatability of the stated procedure, it was necessary to apply the CBD technique to obtain high-quality ZnS films [129]. All researchers who want successful outcomes should consider the above aspects (Section 3.4.1, Section 3.4.2, Section 3.4.3, Section 3.4.4, Section 3.4.5, Section 3.4.6, Section 3.4.7, Section 3.4.8 and Section 3.4.9) that might affect results.

#### 3.4.10. Dopant Concentration Influence on ZnS Thin Film Properties

**Figure 14:**
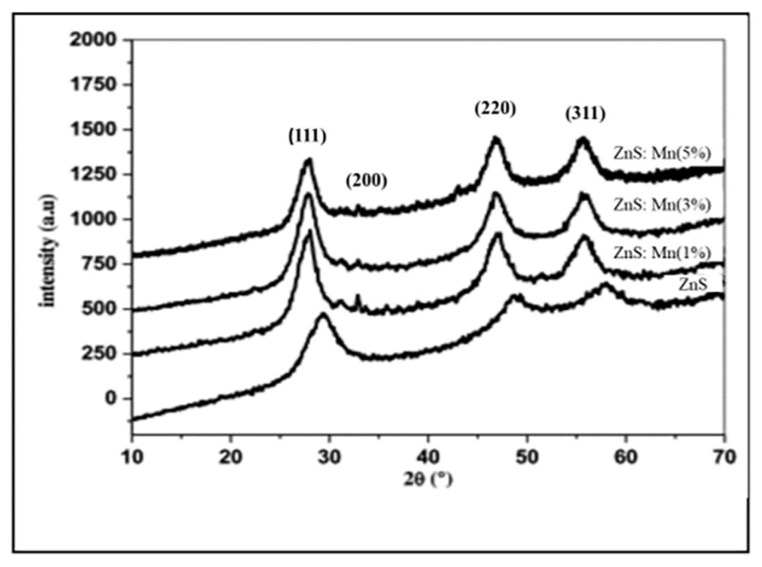
X-ray diffraction data (JCPDS card no: 01-080-0020) of ZnS: Mn thin films with various Mn concentrations (0–5%) by CBD. Reproduced with permission from *Optik* **2017**, *136*, 362–367. Copyright © 2017, Elsevier [132].

**Figure 15:**
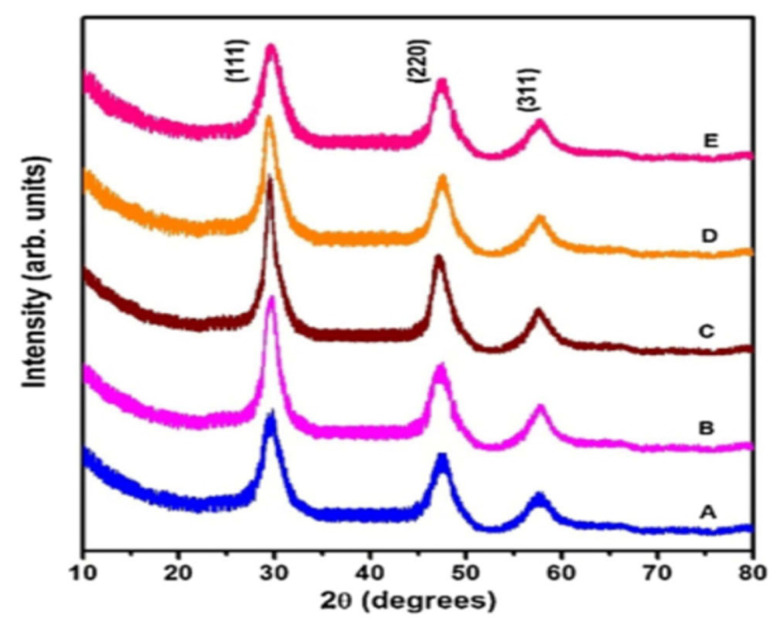
X-ray diffraction patterns (JCPDS Card no. 36-1450) of ZnS thin films with Mn-doping of (A) 0%, (B) 3%, (C) 6%, (D) 9%, and (E) 12%. Reproduced with permission from *Optik* **2017**, *130*, 608–618. Copyright © 2017, Elsevier [136].

CBD is gaining popularity as a relatively straightforward and cost-effective technique. Nevertheless, dopant ions have to be added to stabilize the ZnS system against environmental impacts, including chemical corrosion, enhancement of the structural, optical, and electrical properties, and expand the applications of ZnS thin films [132,133]. It might allow scientists to achieve desirable properties, including excellent optical absorbance, tuning the structure, wide or narrow band gap, variable emission color, ferromagnetism, etc., [55,56,57,134]. For example, in solar energy applications, it is essential to modify its energy levels to absorb solar energy. Doping can introduce intermediate-energy band gap values. Intermediate-energy levels or impurities levels can alter the electronic structure and energy-level transitions [135]. The recent production of doping, e.g., manganese (Mn) [132,136], copper (Cu) [133], iron (Fe) [137], nickel (Ni) [138], cobalt (Co) [139], indium (In) [134], and aluminum (Al) [140] into ZnS thin films utilizing CBD will be covered in this section. Talantikite-Touati et al. [132] synthesized Mn-doped ZnS thin films (at concentrations of 0%, 1%, 3%, and 5%) using CBD (in an alkaline bath, pH = 12.83). The films were deposited on the glass. According to the XRD pattern (see Figure 14), all of the films were crystalline and exhibited a ZnS cubic structure. The crystallite size of the ZnS thin film was larger than that of the ZnS: Mn thin films due to internal strain. The Mn doping boosts the transmittance, with values ranging from 50 to 80% in the visible spectrum. Mn doping enhances the optical characteristics of thin films by 30%. The best transmittance value was obtained for the film with 5% of Mn doping. The values for the band gap were between 3.43 and 3.75 eV. The band gap of thin films reduced as the Mn concentration increased. The band gap of ZnS thin film (3.75 eV) was greater than the 3.68 eV reported for ZnS bulk material. Babu et al. [136] synthesized Mn-ZnS thin films using CBD. The Mn concentration was between 0 and 12%. XRD patterns revealed that Mn-doped ZnS films were crystalline and exhibited a cubic structure (see Figure 15). The grain size of the films increased up to 6% of Mn. After Mn concentrations higher than 6%, the grains tend to dissolve and the film surface becomes porous. The band gap energy varied from 3.68 eV to 3.81 eV with increasing Mn doping. Horoz et al. [135] demonstrated a larger absorption of ZnS: Mn into the visible range improved conversion efficiency due to Mn^2+^ internal-energy transitions to the ZnS. Their study revealed that intermediate-energy levels in wide-band gap widen the absorption window into the visible range to improve solar cell device performance.

Aghaei et al. [133] utilized CBD to produce Cu^2+^ ions (at concentrations: 0.0008, 0.04, and 0.75) doped with ZnS thin films. XRD showed that all Cu: ZnS films were crystalline and formed a cubic zinc blend structure. Increasing the Cu: Zn molar ratio increased the film’s grain size (approximately 100 nm). The band gap energy of Cu:ZnS thin films reduced from 3.84 to 3.64 eV when the molar ratio increased. Transmittance of the films was around 50–70%. The sharp increase in transmittance spectra from 310 nm to 340 nm indicated the homogeneous and compact crystal structure in Cu: ZnS thin films. PL intensity was strongly dependent on dopant Cu concentration and increased significantly with the precursor solutions’ Cu:Zn molar ratio, achieving the highest when the ratio was 0.04:100. The Cu: ZnS films may be used in optoelectronic devices, such as light-emitting diodes.

Akhtar et al. [137] deposited nanocrystalline Fe-doped ZnS thin films on glass substrates using CBD. Fe concentrations were 0–15.62%. All films formed a cubic zinc blend phase structure. SEM showed particle sizes of undoped and Fe-doped ZnS thin films were 65–150 nm. The magnetic study demonstrated that all Fe-doped ZnS thin films exhibit ferromagnetism at room temperature. Akhtar et al. [138] synthesized nickel-doped ZnS thin films onto glass substrates by CBD. According to XRD analysis, the undoped and 6.25% Ni: ZnS thin films exhibited a cubic phase structure. The average particle diameter of the films was 80 nm and Ni-doped ZnS thin films resulted in ferromagnetism at ambient temperature. Akhtar et al. [139] examined Co-doped ZnS thin film using CBD. All ZnCoS thin films were crystalline. After Co doping, the lattice parameter of the films was lower (5.382 to 5.306 Å) than undoped ZnS (5.406 Å). Co concentration slightly increased the band gap energy of the films, which averaged 3.6 eV. The transmittance of ZnS thin films reduced as Co content increased. The observed transmittance ranged between 60 and 80%. Doping concentration increases luminescence centers, which increase green emission PL intensity at 510 nm. Saturation magnetization increased with increasing in Co concentration. Based on half-metallic and the ferromagnetic characteristics of Ni or Fe or Co-doped ZnS [137,138,139], the films might be utilized in spintronic devices.

Jrad et al. [134] prepared indium-doped zinc sulfide (ZnS: In) thin films with In concentrations ranging from 0 to 10% by CBD. The ZnS bath solution was retained at 80 °C and the deposited time of the films was 90 min. The XRD peak intensity (111) of the films increased with increasing In dopant concentration up to 6%. After In concentration was above 6%, the XRD peak intensity decreased indicating a lack of film crystallinity. The band gap energy varied between 3.70 and 3.76 eV for dopant In concentrations ranging from 0 to 10%. The dopant concentration had a low influence on the band gap energy. In addition, the transmittance values (50 and 70%) and reflectance values range from 20 to 40% for all ZnS: In thin films allowing the films to be used as a buffer or an optical window layer in solar cells.

Maria et al. [140] deposited aluminum-doped ZnS (ZnS:Al) thin films on glass using CBD. Al was utilized at a concentration of 0 to 18 weight percent (wt. %). ZnS and ZnS: Al thin films were deposited at 85 °C for 3 h. XRD showed all ZnS: Al thin films exhibited hexagonal wurtzite crystal structures. FESEM showed that AI concentrations of 12 and 18 wt% exceed ZnS’s solubility limit. ZnS: Al films had a thinner thickness (127.61–221.71 nm) than the thickness of the ZnS film, which was 230.27 nm. The XPS spectra reveal the presence of Zn, S, O, C, and Al in the ZnS:Al film. ZnS:Al film with 6 wt.% increased transmittance from 70% to 80%. Through increasing the Al doping percentage, the band gap values reduced from 3.71 eV to 3.52 eV. The carrier concentration varied from $\left(-1.82\times 10^{17}\mathrm{to}-3.13\times 10^{17}{\mathrm{cm}}^{-3}\right)$ and resistivity varied from $2.5810^{5}\mathrm{to}\;1.2510^{5}\;$Ω-cm.

In this section, it is demonstrated that substantial research has been conducted worldwide to analyze the properties of un-doped and doped ZnS thin films using CBD. The role of dopant in ZnS thin film [132,133,134,136,140] contributes to the varied band gap energy, which may be important in the design of a suitable buffer layer for thin film solar cell manufacturing.

## 4. Applications of ZnS Thin Films

Controlling the variables of the CBD (see Section 3) will result in high-quality ZnS films with broad application potential. ZnS attracted considerable interest due to its superior optical and electrical features. Through its unique properties, ZnS had emerged as a promising contender for a variety of applications: photonics, field emission devices, electroluminescence devices, lasers, infrared windows, display technologies, biological devices, nontoxic sensing, optoelectronic devices, and is crucial for buffer layer solar cells [1,10,28]. Nakada and Mizutani [37] and Hariskos et al. [38] discovered that the CBD-ZnS buffer layer on CIGS had an efficiency of 18.1% and 18.6%, respectively.

**Figure 16:**
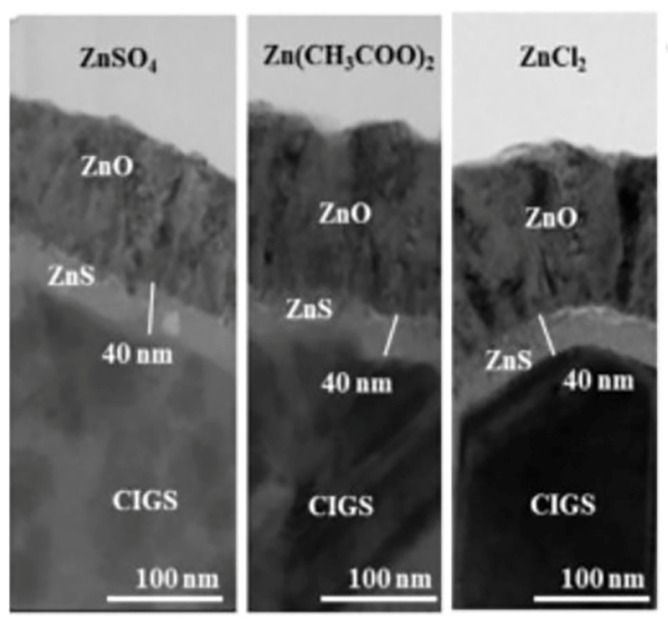
A cross-sectional scanning electron microscope (SEM) of a CIGS thin film solar cell. Reproduced with permission from *Applied Surface Science* **2018**, *432*, 250–254. Copyright © 2018, Elesevier [11].

ZnS can be used as a buffer layer to the $\mathrm{Cu}\left(\mathrm{In},\;\mathrm{Ga}\right){\mathrm{Se}}_{2}$ (CIGS)-based thin film solar cells as shown in Figure 16. This thin buffer layer is utilized to prevent diffusion during deposition operations and improve cell stability [141]. CIGS thin film solar cells are highly efficient photovoltaic (PV) technologies [142]. The CIGS thin-film solar cell with the highest efficiency to date is 23.35% in 2019 [143]. In 2017, CIGSSe established a record for cell efficiency with a maximum of 22.9% [144]. Nakumra et al. [143] utilized double buffer layers with superior characteristics and no Cadmium instead of CdS buffer layers for CIGSSe. Otherwise, Kato et al. [144] utilized the CdS for the CIGSSe buffer layer. Several contemporary record cells had poisonous CdS buffer layers, rendering industrial manufacture and sale in certain locations unfeasible [143]. ZnS utilized alternative buffer layer materials devoid of Cd. The superior efficiency is attributable to the band gap energy (E_g_) of cadmium-free double buffer layers (CBD-ZnS), which is around 3.8 eV [48], which is significantly more than in CdS (2.42 eV) [143]. Subsequently, higher blue light enters the CIGS absorber layer increasing the short-circuit current density (Jsc) [48]. Furthermore, due to exposure to hazardous risks associated with the manufacture and usage of CdS, researchers have focused on the creation of Cd-free buffer layers [1]. Therefore, investigating the prospect of a CBD ZnS-based buffer layers appears fascinating [48].

Present energy demand is rising, and industry relies on irreversible sources. Long-standing global energy concerns have led to the pursuit of clean, renewable energy sources. The renewable energy perspective converts solar energy into electricity making it a potential technology [141]. Future technologies will require eco-friendly, cheaper materials. Thin film photovoltaics are, therefore, predicted to gain popularity. Also needed are cheaper and more energy-efficient techniques. CBD-ZnS films will certainly get greater attention. Bhattacharya and Ramanathan [145] reported that the conversion efficiency of ZnO/ZnS/CIGS solar cells was 18.6%. However, ZnS films had a resistance of roughly 10^7^ Ωcm [140]. Solar cell buffer layers cannot withstand high resistance. ZnS films must be adequately doped because doped films have distinct chemical and physical properties compared to undoped ZnS. Doped transition metals (Mn, Cr, Co, Fe, and Ni) enhanced visible light absorption [140]. Doping nanostructures with trivalent metal cations (Al and In) change their optical and photoluminescence properties [140].

Using the CBD method, homogenous ZnS thin films with improved structural and optical properties have indeed been produced over recent times. CIGS solar cells can use ZnS films with superior optical properties [141]. Solar cells, diode lasers, and optoelectronic devices may potentially utilize higher-quality CBD-ZnS films [43].

## 5. Limitations of CBD Method and Recommendations

Based on the outcomes of the review, this section summarises the issues and concerns that have been identified. Taking on these difficulties will improve the CBD method for producing ZnS thin films. There are a few restrictions/limitations related to CBD techniques [43].

Researchers should use precaution while selecting a substrate that will not react with the precursor solution. SLG or ITO/FTO-coated glass can be employed as the substrate during deposition.

The solubility products of zinc sulfides are typically quite small ~$K_{\mathrm{sp}}$ = 10^−24.7^. The concentration of free Zn^2+^ ions in the CBD solution should be controlled via precipitation throughout depositing. For example, ${\mathrm{ZnSO}}_{4}$ separates into the ions Zn^2+^ and ${\mathrm{SO}}_{4}^{2-}$. The dissolution caused by thiourea: $\mathrm{SC}{\left({\mathrm{NH}}_{2}\right)}_{2}+{\;\mathrm{OH}}^{-}\to {\;\mathrm{SH}}^{-}+{\;\mathrm{CH}}_{2}N_{2}+H_{2}O$ and ${\mathrm{SH}}^{-}+{\;\mathrm{OH}}^{-}$↔ $S^{2-}$+${\;H}_{2}O$. Finally, the bath solution turns into Zn^2+^+ S^2−^ ↔ ZnS [146]. Due to its low solubility, however, ZnS produced by this direct reaction precipitates onto the exposed surface (homogeneous process). Additionally, zinc hydroxide ($\mathrm{Zn}{\left(\mathrm{OH}\right)}_{2}$) precipitation is typical during CBD-grown ZnS [147]. This film would result in low optical transmittance because of its rough topology [146]. $\mathrm{Zn}{\left(\mathrm{OH}\right)}_{2}$ must be minimized to get a high-quality zinc sulfide film. This issue can be resolved by employing the proper complexing agent, which discharges tiny concentrations of ions after the complex ion dissociation and equilibrium constant. The popular complexing agent in CBD baths is ammonia and hydrazine. Ammonia offers an adequate alkaline medium for zinc complex ions, whereas hydrazine can promote film ZnS incorporation and aids in the decrease of hydroxide concentrations [146,147].

CBD solution is usually disposed of after each deposition. Filtering and reacting the precipitate using acids and perhaps other chemicals can obtain starting material for subsequent deposition.

If a researcher performs multilayer CBD depositions, unwanted interactions between previously deposited layers and the deposition solution may occur. Researchers must choose the layering sequence to address this challenge [43].

The desired growth of film and thickness cannot be automatically performed during deposition. The most essential issue for cells with ZnS buffer layers is to regulate ZnS thickness [147]. However, CBD is a promising method for controlling ZnS film thickness and crystallinity. To apply ZnS thin films in buffer layers, the film thickness must be optimized. ZnS film thickness must be 60–73 nm to minimize reflectance at 550–700 nm. With minimal reflectance, the optimum buffer layer can be obtained [76]. If a researcher has to control the film’s thickness, variables, including the stirring rate, the duration of time, and the bath temperature, can be considered.

## 6. Summary and Conclusions

This paper provides an overview of CBD-ZnS thin films as well as numerous parameters that affect their properties and quality. Scopus trends for the second week of November 2022 indicate that CBD ZnS thin film development is gaining momentum and expanding. This is evidenced by the increase of 1560 publications of CBD ZnS thin film from 2011 to a total of 446 publications from 1985 to 2010. CBD is an excellent approach for the ZnS film deposition as it represents the most feasible, reliable, simple, and cost-effective way to produce a thin film in vast areas at near room temperature. There are reportedly three primary deposition processes involved in the CBD technique: ion by ion, cluster by cluster, and a mixed mechanism. Typically, the time of deposition and the nucleation processes determines which mechanism is preferable.

The CBD parameters, such as complexing agent, zinc salt, [Zn]/[S] ratio, stirring speed, humidity, deposition temperature, deposition time, pH solution, precursor types, and annealing, influence the properties of ZnS films. The following is a summary of the most significant findings from this overview of CBD parameters:

Complexing agents affect ZnS thin film growth, homogeneity, and secularity in CBDs. ZnS thin films generated without non-toxic complexing agents have a slow growth rate, a rough morphology, and a discontinuous microstructure. Ammonia and hydrazine are the most commonly used complexing agents for CBD-prepared ZnS films. Hydrazine hydrate smooths the film.

CBD’s reactant concentration ratio [Zn]/[S] in the deposition fluid governs the ZnS thin layer’s physical and chemical properties. Modifying the reactive solution composition can influence the rivalry between homogeneous and heterogeneous nucleation processes to enhance thin film formation.

The stirring effect on ZnS solutions in CBD yields contradictory results in the literature. In general, stirring is beneficial because it promotes particle interaction and introduces solvent components to the solution. The solution became clearer as it was stirred. It was proposed that the ZnS were more densely coated to the glass substrate as the rate of stirring increased (from 300 rpm to 1200 rpm).

The CBD process can be conducted in either a closed reaction vessel (hermetic CBD system) or an open reaction vessel (open CBD system). Hermetic CBD prevents evaporation and environmental interference, whereas humidity disrupts gas–liquid interaction in open CBD systems. The hermetic CBD ZnS film demonstrated superior transmission and morphology in comparison to the open CBD samples. Hermetic CBD is favored for manufacturing an impurity-free thin film.

Researchers generally examined ZnS thin films deposited at temperatures ranging from 60 to 95 °C. The temperature of deposition can be employed to regulate film thickness. As deposition temperature rises, the thickness of ZnS thin films is altered.

CBD-ZnS’s growth, structural, and optical properties depend on the deposition time. CBD ZnS film synthesis began 15 min after the reactants were combined. Various studies have reported varying preparation periods in minutes or hours for ZnS thin films. Increases in deposition temperature control thickness and growth rate. The undesirable ZnO phase disappears with longer deposition times. A prior study found that ZnS growth on glass after 90 min of reaction was more uniform and had good transmission (80% in the spectral region of 300 to 800 nm). Short-circuit current increases with shorter deposition times.

Reaction mixture pH affects thin film formation. The formation of superior thin films requires precursor solutions to include hydroxyl ions (OH^−^). The acidic solution (pH 5) contained Zn^2+^ ions and inadequate circumstances for ZnS film production. Between pH 6 and 10, undesirable intermediate complexes and precipitates are produced but no ZnS. When the pH of the chemical bath is greater than 10, ${\left(\mathrm{Zn}{\left(\mathrm{NH}\right)}_{3}\right)}_{4}^{2+}$ is more likely to form, which is the most desired ZnS product. A pH range of 10 to 12 is optimal for producing high-quality CBD-ZnS films.

Multiple studies have revealed that chemical influences, such as Zn precursor, play an important part in the formation mechanism and physical properties of ZnS thin films. On CBD-ZnS thin growth, the following Zn precursor salts were used: $\mathrm{Zn}{\left({\mathrm{CH}}_{3}\mathrm{COO}\right)}_{2}.2H_{2}O,$ $\mathrm{Zn}{\left(\mathrm{Ac}\right)}_{2},$ ${\mathrm{ZnCl}}_{2}$, ${\mathrm{ZnI}}_{2}$, $\mathrm{Zn}{\left({\mathrm{NO}}_{3}\right)}_{2.}4H_{2}O$, ${\mathrm{ZnSO}}_{4}.7H_{2}O$, and ${\mathrm{ZnSO}}_{4}$. According to current updated research, ${\mathrm{ZnSO}}_{4}$ was discovered as a CBD preferable precursor material for ZnS production.

The annealing factor influences the structural, morphological, optical, and electrical properties. High-temperature annealing is necessary to enhance film crystallinity. Different films necessitate heat treatment at 200–550 °C. Annealing eliminates disturbing surface particles, smoothing films. Annealing ZnS film under vacuum, $N_{2}$, and $N_{2}+{\;H}_{2}$S atmosphere was studied to eliminate the ZnOH phase and increase the crystallinity. The best environment for high-crystallinity ZnS thin films is $N_{2}+{\;H}_{2}$S. This is due to the continuous supply of S during the annealing process.

Doping ZnS thin films with transition metal ions (Ni, Fe, Mn, Co, and Cu) and trivalent metal cations (Al and In) have garnered attention as a way to achieve and tune properties, such as high optical absorption, high transmittance, narrow or wide band gap energy, tunable emission color, ferromagnetism, etc. Precisely controlling doping in nanocrystals can facilitate the synthesis of functional materials exhibiting potentially desirable features for practical uses, such as solar cells, spintronics, etc.

Consequently, numerous CBD parameters influence the morphology, structure, optical, and electrical properties of ZnS films. All researchers who desire successful outcomes should be mindful of all potential influencing parameters. Although it has been demonstrated that CBD-prepared ZnS thin films have improved properties, there are still concerns that require additional exploration. It is crucial to take precautions when selecting a substrate that can react with the precursor solution. The solubility products of zinc sulfides are normally quite small $K_{\mathrm{sp}}$ = 10^−24.7^, and ZnS precipitates onto the exposed surface as a result of this direct interaction. Utilizing a complexing agent that releases modest amounts of ions in line with the complex ion dissociation and equilibrium constant is needed to address this problem. CBD liquids are eliminated following each deposition, hence the precipitation might be filtered and treated using acids and perhaps other chemicals. ZnS thickness control is the most important factor for cells with ZnS buffer layers. If a researcher must manage the thickness of the film, parameters, such as stirring rate, time, and bath temperature, can be assessed. The issues should be resolved shortly due to ZnS films have the potential to serve multiple purposes in a variety of innovative solar cell applications. ZnS is an environmentally safe compound with a higher energy band gap (3.7 eV) than CdS (2.4 eV) that can boost short wave absorption, replace CdS, and substitute Cd-free buffer layer material for CIGSSe thin films. Intending to produce CBD-ZnS that could boost CIGS device efficiency by 20% [49,50], there is still a great deal to discover about the production of optimized ZnS using CBD and its impact on new solar cells. Therefore, numerous CBD techniques and modifications are required to enhance the good features of solar cell materials. The outcomes of the study could provide useful insights and contributions to the synthesis of ZnS thin films utilizing CBD. To obtain the optimal qualities of the ZnS thin film, it is suggested that in-depth research and reviews be made on the parameters of CBD.

## Figures and Tables

**Table 1:** Properties of Zinc Sulfide (ZnS) [9,42].

	Properties	Characteristics
Physical	Odour	Sulfurous odour
	Solubility in water	Insoluble
	Appearance	White-greyish to yellow powder
Chemical	Empirical formula	ZnS
	Molar mass	97.46 g/mol
	Lattice constant	5.4093 Å
	Crystal structure	Cubic
	Group	Zinc-12
Mechanical	Density	4.079 g/cm^3^
	Poisson’s Ratio	0.27
	Flexural Strength	103 MPA
	Modulus elasticity	75 GPA
	Boiling point	1185 °C
	Melting point	1850 °C
Electric	Electronic configuration	Zinc: [Ar]3d10^4^S^2^
	Dielectric constant	8.9
	Band gap	3.54 eV
	Hole mobility	5 cm^2^/Vs
	Electron mobility	180 cm^2^/Vs
Thermal	Heat of information	477 KJ/mol
	Heat of fusion	390 J/g
	Thermal conductivity	25.1 W/mk
	Specific heat capacity	0.472 J/g°C
	Thermal coefficient of expansion	6.36 µm/m °C
Optical	Refractive index	2.356

**Table 2:** Primary factors and their impact on diverse CBD-ZnS thin films [43,81].

Parameters of CBD	Affected Properties of ZnS Thin Films
Complexing agent	Crystalline characteristics, thickness, morphology, surface roughness, and optical transmittance
Zinc salt and [Zn]/[S] ratio	Crystalline characteristics, film growth, and morphology
Stirring speed	Film growth rate, thickness, and surface roughness
Humidity	Crystalline characteristics, morphology, and optical transmittance
Deposition temperature	Morphology, pH of the solution, thickness, and optical transmittance
Deposition time	Crystalline characteristics, thickness, and band gap
pH value	Crystalline characteristics, surface morphology, optical transmittance, film growth rate, and band-gap
Precursor types	Morphology, optical transmittance, and band gap
Annealing (environmental and temperature effect)	Crystalline characteristics, morphology, and optical transmittance

## Data Availability

Not applicable.
